# Cheater suppression and stochastic clearance through quorum sensing

**DOI:** 10.1371/journal.pcbi.1010292

**Published:** 2022-07-28

**Authors:** Alexander S. Moffett, Peter J. Thomas, Michael Hinczewski, Andrew W. Eckford

**Affiliations:** 1 Department of Electrical Engineering and Computer Science, York University, Toronto, Ontario, Canada; 2 Department of Mathematics, Applied Mathematics, and Statistics, Case Western Reserve University, Cleveland, Ohio, United States of America; 3 Department of Physics, Case Western Reserve University, Cleveland, Ohio, United States of America; University of Cambridge, UNITED KINGDOM

## Abstract

The evolutionary consequences of quorum sensing in regulating bacterial cooperation are not fully understood. In this study, we reveal unexpected effects of regulating public good production through quorum sensing on bacterial population dynamics, showing that quorum sensing can be a collectively harmful alternative to unregulated production. We analyze a birth-death model of bacterial population dynamics accounting for public good production and the presence of non-producing cheaters. Our model demonstrates that when demographic noise is a factor, the consequences of controlling public good production according to quorum sensing depend on the cost of public good production and the growth rate of populations in the absence of public goods. When public good production is inexpensive, quorum sensing is a destructive alternative to unconditional production, in terms of the mean population extinction time. When costs are higher, quorum sensing becomes a constructive strategy for the producing strain, both stabilizing cooperation and decreasing the risk of population extinction.

## Author summary

Quorum sensing is a process through which bacteria can regulate gene expression according to their population density. The reasons for why bacteria use quorum sensing to regulate production of “public goods”, biochemical products that benefit nearby bacteria, are not entirely clear. We use mathematical modeling to explore how quorum sensing compares to other strategies for controlling production of public goods, namely unconditional production independent on population density, in small populations of bacteria where the random nature of growth is significant. Our model captures both how likely “cheater” strains, which do not produce public goods but benefit from them, are to take over a population and how long on average the population will last before going extinct. We find that depending on how expensive public good production is and how critical public goods are for growth, quorum sensing can decrease or increase the mean time to extinction compared with unconditional production, while always reducing the likelihood of cheaters taking over. Our results could have important implications for the growth of bacterial infections, for example *Pseudomonas aeruginosa* infections of the lungs of cystic fibrosis patients.

## Introduction

Cooperative behavior is widespread in bacteria [1], including coordinated swarming and public good production. These behaviors are often regulated through quorum sensing (QS) [2], in which individual bacteria produce and export small molecules called autoinducers (AI). When AI molecules accumulate to a sufficiently high concentration in the environment, and consequently within the bacteria producing them, they activate operons controlling the expression of genes critical for cooperation. While the biochemistry of some QS systems is well understood [2], QS is sensitive to a number of factors [3–5] and there are many proposed biological functions of QS which have been the subject of debate [6–9].

Evolutionary questions concerning QS function have largely focused on the ability of QS-controlled cooperation to combat invasion by non-cooperating cheaters in bacterial populations [10, 11]. The question of how cooperation can evolve and be maintained in populations is a general problem in evolutionary biology [12], and the role of QS in the evolution of bacterial cooperation is of great interest in understanding bacterial social interactions. A number of possible resolutions to the problem of social cheaters in bacterial public good production include punishment of cheaters [13], dispersal into subpopulations [14], and the use of QS to regulate cooperation [1, 8, 15]. Regulation of public good production through QS has been shown to reduce the ability of cheaters to invade a population of producers [16].

The role of QS in maximizing population growth in the absence of cheaters has also been investigated as a rationale for QS-control of public good production [17]. Because public goods produced by bacteria can have density-dependent fitness benefits [18], regulation of public good production based on population density can be seen as an optimal control solution to maintaining a maximal population size balancing metabolic costs and benefits [19, 20]. By maintaining a maximal population size, a population also maximizes its mean time to extinction, which is especially important with the possibility of unpredictable environmental changes.

Considering the threat of both cheater invasion and the onset of harmful environmental conditions that could lead to population extinction, there is a tension in the degree to which public good production is regulated. Unconditional public good production appears to be a self-defeating strategy, where non-producing cheaters will arise by mutation and reap all the benefits of public goods without experiencing any of the associated production costs. At the other extreme, a strategy where no public goods are produced (which could be a result of a successful cheater invasion of a cooperating population) should be vulnerable to extinction when the public goods are essential for growth or reducing the likelihood of death. The idea that quorum sensing is a moderate strategy between these two extremes has been explored previously [21]. However, to our knowledge, a full analysis of this tension explicitly considering both cheater fixation probability and mean population extinction time has not been carried out. What are the effects of QS-mediated regulation of public good production in terms of cheater suppression and overall population robustness? Does QS always protect against cheaters while also increasing long-term viability of the population?

In this work, we explore the effects of QS on cheater fixation and mean population extinction time in a simple birth-death model of mixed producer-cheater populations. Our model sacrifices much of the complexity of QS [3–5] in order to provide more general insight into how QS strategies influence the eco-evolutionary dynamics of bacterial colonies. We compare the QS strategy of public good production with an “always on” (AO) strategy, meaning that each producer cell unconditionally produces public goods at the maximum rate. Our models reflect bacterial populations where demographic stochasticity is an important factor in population dynamics. The role of demographic stochasticity in bacterial populations has been explored in past work [22–25], and is particularly important when populations are divided into subpopulations.

An important example of subdivided populations is *Pseudomonas aeruginosa* infections in the lungs of cystic fibrosis (CF) patients [26]. Bacteria often form small, dense biofilm aggregates in infections [27]. In CF lung infections, *P. aeruginosa* forms aggregates of at most ∼1, 000 cells [28], suggesting that demographic noise is an important factor. While biofilms are known to protect bacteria from the effects of antibiotics [29], the possibility of “stochastic clearance” where relatively small bacterial populations go extinct at sub-minimum inhibitory concentrations of antibiotics [24, 30] suggests that extinction is a real possibility faced by *P. aeruginosa* aggregates in the presence of stressors such as antibiotics. Furthermore, while *in vitro* experimental evidence suggests that QS induction can occur within these aggregates [31], QS induction is unlikely to occur between distinct aggregates [32]. This evidence provides justification for our focus on single well-mixed sites in QS, an important first step towards a more realistic model incorporating interactions between separate aggregates and accounting for heterogeneity within single aggregates. We use the QS system controlling production of proteases by *P. aeruginosa* as inspiration for our model, where public goods increase the growth rate of all individuals while leaving death rates unaffected. Without the ability to produce proteases, *P. aeruginosa* starves when proteins are the sole source of carbon and nitrogen [33]. However, with the ability to produce and export proteases into their environment, *P. aeruginosa* can grow using the oligopeptides that proteases produce by cleaving exogenous proteins.

We find that while QS decreases cheater fixation probability for all examined public good costs and constitutive growth rates (growth rate in the absence of public goods), the population mean extinction time is only increased by QS for a well-defined set of cost-growth rate pairs. When mean extinction time is decreased by QS, there is an increased risk of stochastic clearance. By “stochastic clearance”, we refer to the phenomenon where a population with a non-negative mean net-growth rate has a finite time to extinction, due to stochastic copy number fluctuations. The cases where QS decreases mean extinction time as compared with an unconditional AO strategy is an example of a weak form of “evolutionary suicide” [34], where QS increases the relative fitness of the cheater strain while the entire population of both producers and cheaters is made more vulnerable to extinction. We call this situation destructive cheater suppression. Only when both the cost of public good production and the constitutive growth rate are large enough is QS a constructive strategy for the producers, in that both cheater fixation probability is reduced and mean extinction time is increased.

## Results

**Fig 1:**
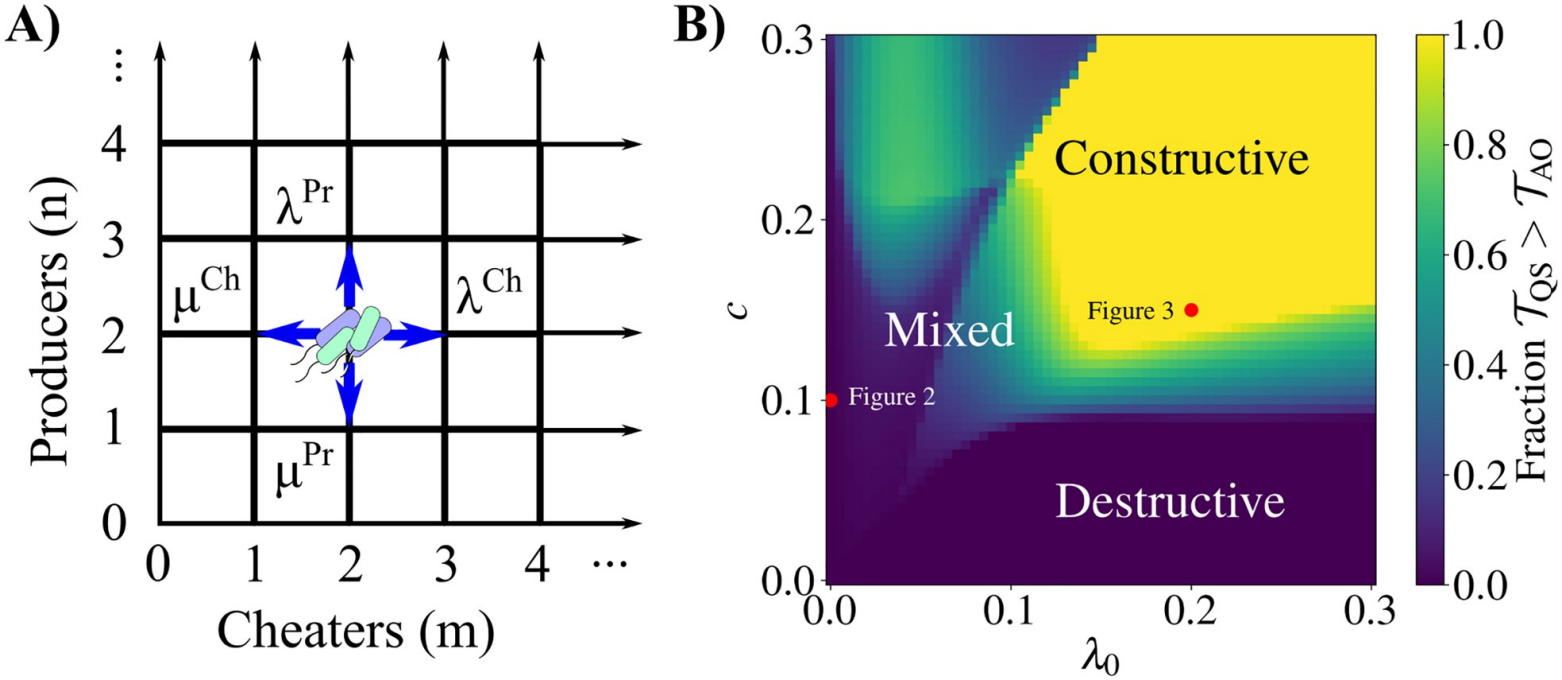
Overview of birth-death model and main results. A) Diagram of the two-dimensional birth-death process describing the dynamics of a bacterial population consisting of public good producers and cheaters. As an example, the population is shown with two producers and two cheaters. All possible subsequent states of the population are indicated, where a single producer or cheater can either arise through binary fission (with state-dependent rates λ^Pr^ and λ^Ch^, respectively) or can die (with state-dependent rates *μ*^Pr^ and *μ*^Ch^). See Eqs 1–4 for the full forms of the birth and death rates. B) Phase diagram describing the fitness gains of quorum sensing (QS) over always on (AO) producers, as a function of constitutive growth rate (λ_0_) and public good production cost (*c*). For each pair (λ_0_, *c*) we calculated the mean extinction time for all (*n*, *m*) pairs satisfying *n* ≥ 0, *m* ≥ 0, and *n* + *m* ≤ 100 with the QS and AO strategies. The reported number is the fraction of these (*n*, *m*) pairs for which the mean extinction time for QS is greater than for AO ($T_{n,m}^{\text{QS}}>T_{n,m}^{\text{AO}}$). In the bottom right-hand region (dark blue) regulating public good production through QS decreases mean extinction time for all (*n*, *m*) pairs, meaning that the AO strategy decreases the risk of population extinction. Here, QS is a destructive strategy. The upper region (yellow) is where QS increases the mean extinction time for all (*n*, *m*), a constructive strategy for the producers. The region labeled “mixed” indicates that QS increases mean extinction time for some (*n*, *m*) pairs while decreasing it for others.

We first briefly introduce the notation used throughout the remainder of this article. The number of producers in the population is written as *n* while the number of cheaters is *m*. When *n* = *m* = 0, the population of bacteria has gone extinct. The cost of public good production is *c* and the per-capita constitutive growth rate (in the absence of public goods) is λ_0_. The per-capita death rate for all individuals is *μ*_0_. The birth rates given *n* and *m* are $\lambda_{n,m}^{\text{Pr}}$ and $\lambda_{n,m}^{\text{Ch}}$ while the death rates are $\mu_{n,m}^{\text{Pr}}$ and $\mu_{n,m}^{\text{Ch}}$, for the producers and cheaters, respectively (Fig 1A). We write the cheater fixation probability as *π*^Ch^, the mean time to cheater fixation as *τ*^Ch^, and the mean time to population extinction as T. The model is fully described in the Materials and Methods section.

The phase diagram in Fig 1B shows the fraction of (*n*, *m*) pairs in the set $\left\{\left(n,m\right)\in Z^{2}\;|\;n,m>0,\;n+m\leq 100\right\}$ in which the mean extinction time of a population regulating public good production through QS is larger than that using the AO strategy, as a function of the constitutive per capita growth rate, λ_0_ ≥ 0, and the cost of public good production, 0 ≤ *c* ≤ 1, representing the fraction of growth rate benefit provided by public goods alone. We first examine two points of interest on this phase diagram, *c* = 0.1, λ_0_ = 0 and *c* = 0.15, λ_0_ = 0.2 as marked in Fig 1B, and then discuss the general features of the phase diagram.

### Quorum sensing is a destructive strategy in the absence of alternative nutrition sources

**Fig 2:**
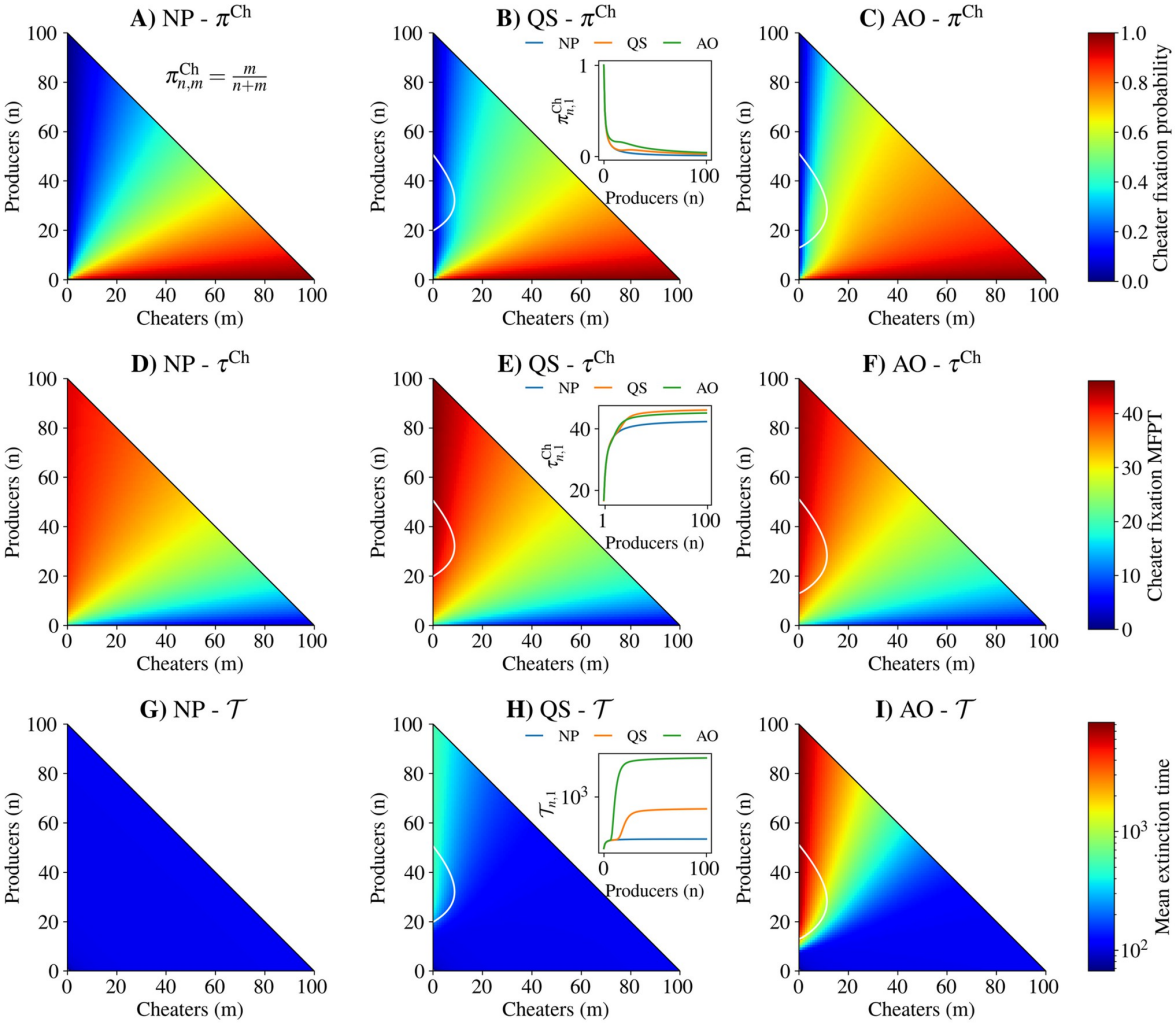
Quorum sensing is a destructive strategy for producers when no alternate energy source is present (λ_0_ = 0) and when public good cost is moderate (*c* = 0.1). Row 1: Cheater fixation probability from an initial population of *n* producers and *m* cheaters for A) no production (NP), B) quorum sensing (QS), and C) always on (AO) strategies. Row 2: Conditional mean first passage time to cheater fixation from initial population structure for D) no production, E) quorum sensing, and F) always on strategies. Row 3: Mean extinction time from initial population structure for G) no production, H) quorum sensing, and I) always on strategies. White traces: zero expected net total population growth contours. Inset plots in B), E), and H) show cheater fixation probability, cheater mean first passage time to fixation, and mean extinction time, respectively, for an initial population with one cheater and *n* producers. See S1 Table for parameter values.

In agreement with previous work [16, 35], we find that in the absence of alternate sources of nutrition (λ_0_ = 0), QS reduces the probability of cheater fixation for a wide range of starting population compositions (Fig 2). We calculate the probability of cheaters fixing (Eq 9) in a small population with starting compositions satisfying 0 < *n*, 0 < *m*, and *n* + *m* ≤ 100. We compare cases where no public good is produced (NP), where public good production is QS-controlled, and where public good production is always on (AO). The NP strategy corresponds to a cheater strain that produces autoinducer. Because there is no constitutive growth rate (λ_0_ = 0), if the NP strategy is used there is no (*n*, *m*) pair for which the net population growth rate is non-negative. On the other hand, for the QS and AO populations, the white lines in Fig 2 represent the total population zero expected net growth contour. Because these lines represent the zero expected net growth rate contours of the entire population, they are not fixed points of the underlying deterministic behavior of the system, which only exist at the origin and at the intersection of the zero expected net growth contours with the producer axis. Thus, there can not be a long-lived population with a non-zero number of cheaters.

With a moderate cost to public good production (10% of the maximum growth rate benefit imparted by public goods), a QS strategy (Fig 2B) increases cheater fixation probability as compared with an NP strategy (Fig 2A) while decreasing cheater fixation probability as compared with an AO strategy (Fig 2C). This can be clearly seen in an invasion scenario with a single cheater present in the population (Fig 2C inset). For any public good production strategy, without any elaborate solutions such as policing [36] it is unavoidable that cheaters will become more likely to fix in a population. However, QS mitigates this possibility as compared with an AO strategy. Both the QS and AO strategies increase the mean time to cheater fixation (Fig 2E and 2F) and the mean time to extinction (Fig 2H and 2I) as compared with the NP strategy (Fig 2D and 2G, respectively) for starting compositions with few cheaters and many producers. Our results match well with stochastic simulations (S1 and S2 Figs).

In order to explore the ecological consequences of public good production for the whole bacterial population, we calculated the mean time to extinction of the population given an initial composition of producers and cheaters. The mean extinction time has been proposed as a measure of bacterial tolerance to antibiotics [30], representing the ability of bacterial populations to persist when confronted with stressors. While QS reduces the probability of cheater fixation as compared to AO, the mean extinction time with QS (Fig 2H) is greatly reduced as compared to AO (Fig 2I). This suggests that with no alternate sources of nutrition, QS increases the relative fitness of producers while decreasing the overall population fitness as compared with AO. This scenario, which we call destructive cheater suppression, could be an example of evolutionary suicide [34] (see Discussion), though in a weak sense because the population becomes more likely to go extinct through fluctuations rather than a non-zero equilibrium population size disappearing [37]. For most, but not all, (*n*, *m*) pairs, the mean extinction time for QS is larger than for AO. This places the system in the “mixed” region of the phase diagram (Fig 1B).

The qualitative differences in cheater fixation probabilities and mean extinction times between QS and AO were preserved for systems with carrying capacities of 200 (S3 Fig), twice the size considered in Fig 2 (see S1 and S2 Tables for the parameters used). While this does not guarantee that the destructive nature of QS in these conditions is independent of populations size, it does demonstrate insensitivity to the overall population size. Given that our model is not parameterized by experimental results, the population sizes we consider do not directly correspond to population sizes in real bacterial colonies. Instead, our model aims to capture key aspects of bacterial population dynamics when demographic noise plays a role, as discussed in the Introduction. The degree of demographic noise is related to the size of a population through the law of large numbers, where the standard deviation in the population size over the mean number of individuals is inversely proportional to the square root of the population size. For this reason we focus on small populations, where demographic noise is relatively large.

### Alternative sources of nutrition enable constructive suppression through QS

**Fig 3:**
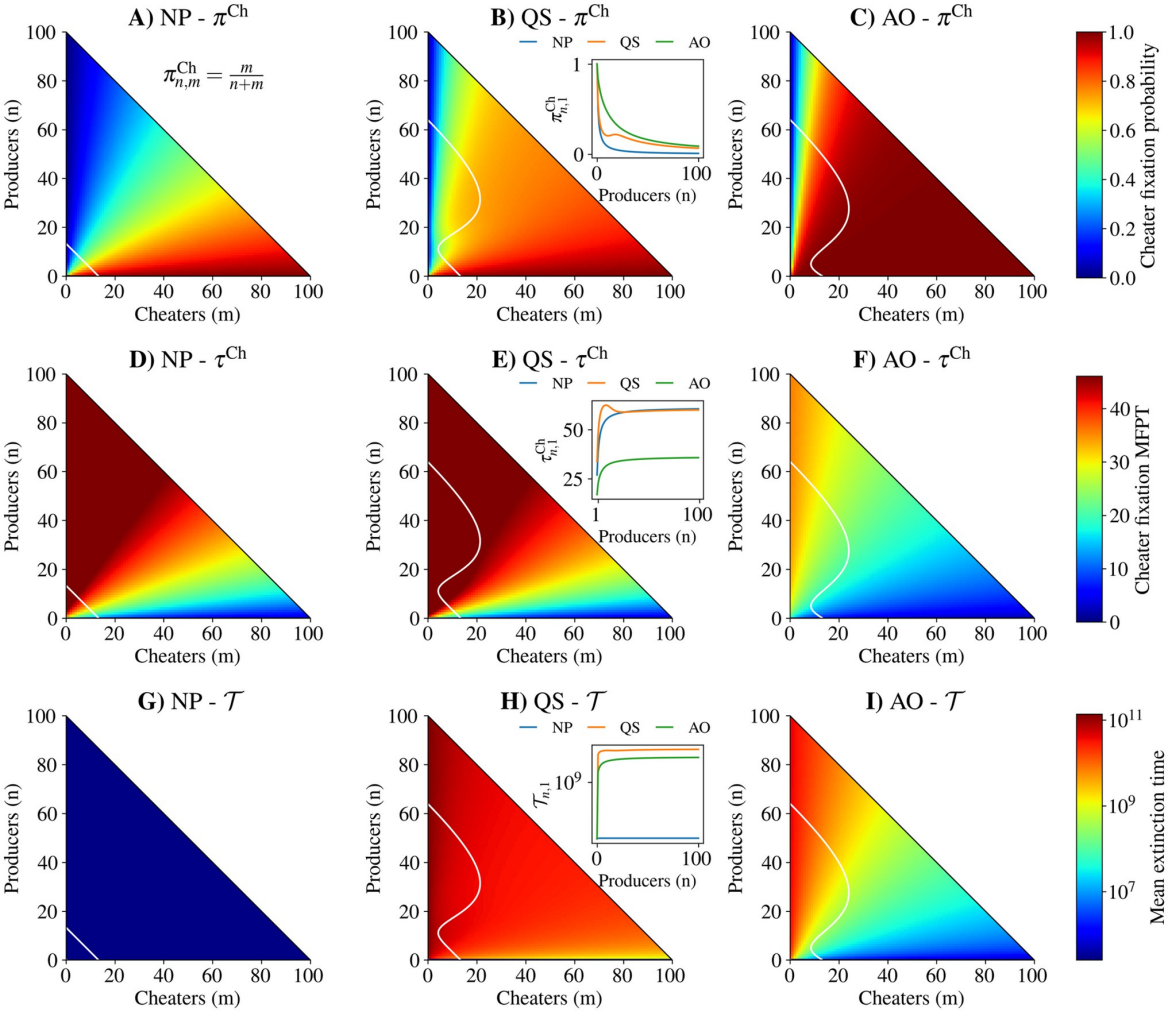
Quorum sensing is a constructive strategy for producers when an alternate energy source is present (λ_0_ = 0.2) and when public good cost is moderate (*c* = 0.15). Row 1: Cheater fixation probability from an initial population of *n* producers and *m* cheaters for A) no production (NP), B) quorum sensing (QS), and C) always on strategies (AO). Row 2: Conditional mean first passage time to cheater fixation from initial population structure for D) no production, E) quorum sensing, and F) always on strategies. Row 3: Mean extinction time from initial population structure for G) no production, H) quorum sensing, and I) always on strategies. White traces: zero expected net total population growth contour. Inset plots in B), E), and H) show cheater fixation probability, cheater mean first passage time to fixation, and mean extinction time, respectively, for an initial population with a single cheater and *n* producers. See S1 Table for parameter values.

Bacterial populations need not rely solely on the growth benefits of a public good. In some cases they may use alternative sources of nutrition which are not directly influenced by public good production. We consider the case in which the constitutive growth rate for individuals with zero public goods present is non-zero but small (20% of the maximal growth rate benefit provided by public goods alone). This non-zero constitutive growth rate allows for a small carrying capacity to appear in the NP case, indicated by the white line in the leftmost column of Fig 3. With the QS and AO strategies, the zero expected net growth contour reflects the NP carrying capacity when the number of producers is low, but increases markedly with more producers. Additionally, more cheaters can be accommodated with more producers present because of the public nature of the fitness benefits provided by producers. As in Fig 2, the only fixed points of the underlying deterministic dynamics are the origin and the intersections of the zero expected net growth contour with the axes.

As in the case of zero constitutive growth, QS (Fig 3B) decreases the probability of cheater fixation over AO (Fig 3C) for a wide range of starting population compositions, while increasing the fixation probability compared to NP (Fig 3A). The improvement of QS over AO can be clearly seen in the cheater invasion scenario in the inset of Fig 3B. However, with non-zero constitutive growth the mean time to cheater fixation for NP (Fig 3D) and QS (Fig 3E) are comparable, while cheaters fix more quickly for AO (Fig 3F). Again, our results match well with simulations (S4 and S5 Figs).

The presence of an alternative source of nutrition renders QS a beneficial strategy for producers (Fig 3) at a moderate public good production cost (15%). QS increases the mean extinction time of the population for all starting population compositions with at least one cheater and one producer over AO (Fig 3H and 3I). Both QS and AO increase the mean extinction time over NP (Fig 3G). From comparison with (Fig 2), it appears that the degree to which growth depends on alternative nutrition sources influences whether QS at moderate costs is destructive or constructive.

As when there is no constitutive growth, the qualitative differences between QS and AO were preserved for systems with carrying capacities of 200 as shown in S6 Fig (see S1 and S2 Tables for parameters).

### Trade-offs between cheater suppression and mean extinction time

**Fig 4:**
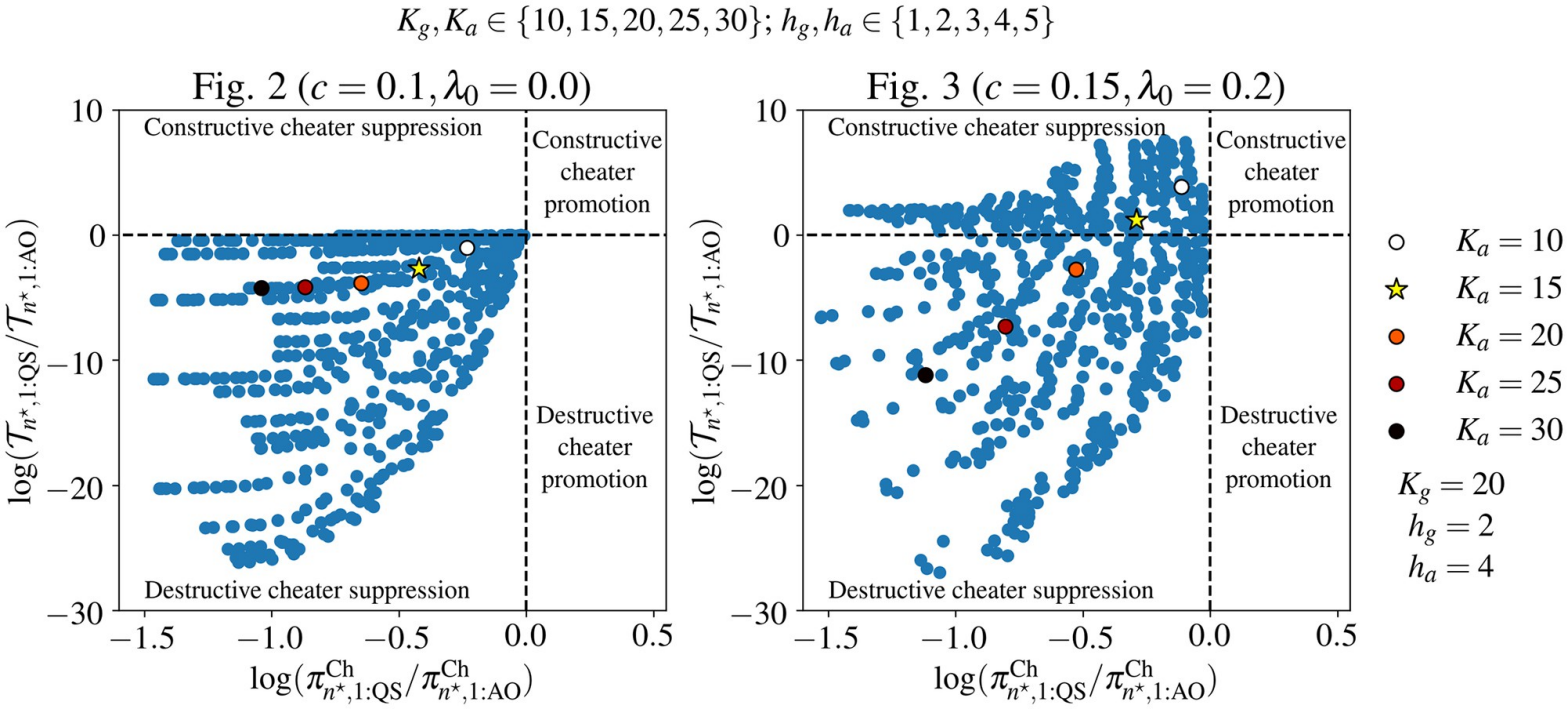
The results of Fig 2 are insensitive to changes in parameters describing public good growth benefit and QS activation (left plot), while the result of Fig 3 are robust to the same parameters, but can be reversed depending on the parameter values (right plot). We calculated the log-ratio of mean extinction times, $\text{log}(T_{n^{⋆},1:\text{QS}}/T_{n^{⋆},1:\text{AO}})$, and the log-ratio of cheater fixation probabilities, $\text{log}(\pi_{n^{⋆},1:\text{QS}}^{\text{Ch}}/\pi_{n^{⋆},1:\text{AO}}^{\text{Ch}})$, for all 625 different combinations of *K*_*g*_, *K*_*a*_ ∈ {10, 15, 20, 25, 30} and *h*_*a*_, *h*_*g*_ ∈ {1, 2, 3, 4, 5}. Each point on the plot represents the results for one of the 625 parameter combinations. The mean extinction times and cheater fixation probabilities were calculated for $n^{⋆}=\text{round}(\frac{\lambda_{0}+g-c}{\mu })$ producers and *m* = 1 cheater, the relevant initial population composition for the case of a single cheater arising by mutation in a population or producers. The highlighted points show how varying *K*_*a*_ alone affects the results (*K*_*a*_ = 15, used in Figs 2 and 3, is indicated by a star), with *K*_*g*_, *h*_*g*_, and *h*_*a*_ fixed as in Figs 2 and 3.

We next examine how varying the parameters controlling public good growth benefit and production affect whether QS is destructive or constructive. Fig 4 shows the effects of different *K*_*g*_, *h*_*g*_, *K*_*a*_, and *h*_*a*_ values on the main results of Figs 2 and 3. QS is always destructive with the λ_0_ and *c* values of Fig 2 (*c* = 0.1 and λ_0_ = 0), while QS could be either constructive or destructive in the case of Fig 3 (*c* = 0.15 and λ_0_ = 0.2). None of the parameter sets we examined for either (*c*, λ_0_) pair fell into the region of cheater promotion, where QS would increase the cheater fixation probability over AO.

The effects of *K*_*a*_ on the success of a single producer in colonizing a habitat and on the probability of a single cheater leading to fixation of cheaters in an established population of producers are shown in S14 and S15 Figs, respectively. This demonstrates the opposing factors in determining the optimal value of *K*_*a*_. Lower values of *K*_*a*_ are beneficial to the growth of pure producer populations, balancing the costs and benefits of public good production, while large *K*_*a*_ values reduce the probability of cheater fixation. This trade-off is reflected in Fig 4 and S16 Fig by the highlighted points on the plots, again demonstrating that the risk of stochastic clearance increases as the risk of cheater fixation decreases for QS compared to AO.

### Autoinducer production by cheaters is a destructive strategy

**Fig 5:**
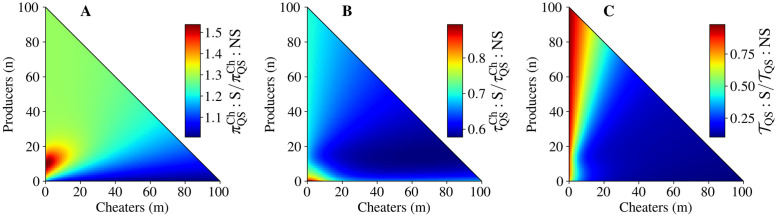
Cheater autoinducer production is destructive in the presence of an alternative source of nutrition (λ_0_ = 0.2). The ratio of A) cheater fixation probability, B) cheater mean fixation time, and C) mean extinction time between QS populations where cheaters signal (indicated by *S*) to where cheaters do not signal (indicated by *NS*). The non-signaling results used in the denominators are the same as those in Fig 3. See S1 Table for parameter values.

To this point, we have assumed that cheaters produce neither public goods nor autoinducer. What are the consequences of cheaters producing autoinducer while still not producing public goods? We performed the same analysis as in the previous sections using the growth rates in Eqs 5 & 6 which reflect equal autoinducer production by producers and cheaters. We compared these results to those presented in Fig 3, where an alternative nutrition source exists and cheaters do not produce autoinducer. Cheater signaling increases the probability of cheater fixation while decreasing the mean time to cheater fixation and the mean extinction time (Fig 5). From the perspective of the cheater population, autoinducer production is a destructive strategy. Even though cheater signaling decreases mean extinction time, this result suggests that signaling cheaters are more likely to be observed in nature than non-signaling cheaters, provided that signaling cheater mutations are at least as likely to occur in a population as non-signaling cheater mutations.

### Producer advantage in the benefits of public goods reduces the advantages of QS

The role of the spatial structure of populations and the diffusive properties of their environments has recently been acknowledged as a possible explanation for why cheaters do not always fix in a population of producers [8]. Essentially, if producers are more likely to be close to other producers, and the benefits of the public good are spatially confined to an area close to a producer, cheaters will be unlikely to benefit from public goods. We examine how this type of advantage to producers effects our results by adding a non-zero constant *a* to the *g* parameter in the producer growth rate (Eq 1), which reflects the possibility that producers benefit more than cheaters from public goods because of their spatial arrangement. We look at how the results of Fig 2 change with increasing *a* (S7 Fig), finding that larger *a* values decrease the benefits of QS as compared with AO both in terms of cheater fixation probability and mean extinction time. With large *a*, there is a permanent growth advantage for producers when public goods are present, so delaying production of public goods also delays the advantage. For *a* = 0.2, QS is not only destructive in that it reduces mean extinction time, but with some initial population compositions it actually increases cheater fixation probability over AO. The results for Fig 3 change in a similar way with *a* (S8 Fig), except that the relative cheater fixation probabilities are less noticeably altered by *a*. Altogether, producer advantage, whether due to co-localization of producers or other mechanisms, shifts QS towards being destructive with respect to AO.

### The destructive or constructive nature of QS depends on cost and constitutive growth

The phase diagram in Fig 1B demonstrates the role of both public good production cost and constitutive growth rate in determining the nature of QS outcomes. At low constitutive growth rates, public good production is essential to the survival of the population. With low λ_0_, QS regulation of public good production moves to the “mixed” region of the phase diagram at relatively low public good costs. This means that whether QS is beneficial to the population depends on the initial composition of the population. For low constitutive growth rates below λ_0_ = 0.1, all values of public good cost investigated lead to a mix of destructive and constructive cheater suppression. With larger constitutive growth rates the transition from destructiveness to constructiveness with increasing cost *c* occurs gradually with the transition to constructiveness completing at *c* = 0.1 to *c* = 0.15, depending on λ_0_. For all (*c*, λ_0_) pairs with *c* > 0, the cheater fixation probability is reduced in QS as compared with AO (S9 Fig).

We show the ratio $T_{QS}/T_{AO}$ for all (*n*, *m*) pairs for two sequences of (*c*, λ_0_) pairs in S10 and S11 Figs. With fixed *c* = 0.2, the ratio $T_{QS}/T_{AO}$ is always highest with intermediate mixtures of producers and cheaters (S10 Fig). When λ_0_ ≈ 0.1, there is a transition from the mixed region to the constructive region, where (*n*, *m*) pairs with few cheaters appear to be slower to change to constructiveness with increasing λ_0_. With fixed λ_0_ = 0.2, there is a gradual transition from destructiveness at low *c* to constructiveness at high *c* (S11 Fig). At moderate costs below *c* = 0.1, QS is destructive, with $T_{QS}<T_{AO}$ for all (*n*, *m*) pairs. However, once the cost reaches 0.15, QS is no longer destructive, as the mean extinction time increases with respect to AO. In the transition range from *c* = 0.1 to *c* = 0.15, the ratio $T_{QS}/T_{AO}$ is highest when the number of producers is low, suggesting that QS is most beneficial with those initial population structures. The QS gain in mean extinction time over AO is further increased by QS at a cost of 0.2, again most drastically when *n* is small.

The phase diagram displays some non-monotonic behavior in *c* and λ_0_, especially in the low λ_0_, high *c* region (Fig 1B). This is a result of calculating the fraction of (*n*, *m*) pairs where $T_{\text{QS}}>T_{\text{AO}}$, so that the phase diagram is a superimposition of results for each (*n*, *m*) pair. If we instead look at the fraction of (*n*, *m*) pairs where $\text{log}(T_{\text{QS}}>T_{\text{AO}})>\delta$ for a tolerance parameter *δ*, we can see that the non-monotonic behavior of the low λ_0_, high *c* region disappears with small, positive *δ* (S12 Fig). This indicates that in this region, the mean extinction times are relatively similar. On the other hand, with negative *δ* the destructive region with low *c* remains, indicating that $T_{\text{QS}}$ is notably smaller than $T_{\text{AO}}$ there. We can see the same patterns by looking at $\text{log}(T_{\text{QS}}>T_{\text{AO}})$ over λ_0_ and *c* for a selection of (*n*, *m*) pairs (S13 Fig). This further reinforces that QS is most constructive with large λ_0_ and *c*.

## Discussion

Our model of competitive growth between bacterial producers and cheaters reveals a number of previously unexplored consequences of quorum sensing. We investigate the case of isolated, single subpopulations of bacteria as a first step towards understanding the impact of QS on bacterial eco-evolutionary dynamics. When public good production allows for metabolism of otherwise inaccessible sources of nutrients, we demonstrate the important role of both the availability of alternative, public good-independent nutrients and the cost of public good production in determining the consequences of quorum sensing. With no alternative nutrients, quorum sensing by producers withholds the only source of nutrients from the whole population, which harms both producers and cheaters in terms of the total population’s mean extinction time. We identify this result as a possible example of demographically stochastic evolutionary suicide [37], as quorum sensing increases the relative fitness of producers while decreasing the mean extinction time of the entire population. If instead there is an alternate energy source, then regulating public good production via quorum sensing reduces the probability of cheater fixation, while in some cases increasing the mean extinction time of the population over the always on strategy and in other cases decreasing the mean extinction time, depending on the public good production cost.

The destructive region in Fig 1 can be understood heuristically by considering that, if the cost of public good production is low, QS-mediated regulation of public good production withholds the fitness benefits of the public good while achieving minimal metabolic savings for the producers. In addition, large alternate sources of nutrition can help to offset the costs of public good production, reducing loss in relative producer fitness due to costs. On the other hand, when public good production is costly, QS-mediated production balances costs and benefits by delaying public good production until the population of producers is large enough to benefit. Thus both public good production cost and constitutive growth rate play an important role in determining the eco-evolutionary consequences of QS-based regulation.

Because we have not explicitly modeled the evolution of the QS strategy, our suggestion that destructive QS is an example of evolutionary suicide requires further analysis. Specifically, the suggestion that QS could evolve when it falls in the destructive phase of Fig 1B is based only on the fact that QS results in lower cheater fixation probability, and hence in larger relative fitness as compared with cheaters, than the AO strategy. Additional work, including explicit modeling of QS evolution, is needed to confirm the connection with evolutionary suicide and would be an interesting direction for future research.

Future experimental work is needed to test the predictions of our model. Experimental techniques such as those used by Coates *et al*. [24] together with genetic manipulation of QS circuitry could provide an avenue for testing our predictions, and would provide invaluable insight into the role of QS in small bacterial populations. Further theoretical work could explore the impact of mutation, migration, and horizontal gene transfer on our results, where a stable heterogeneous steady state could be achieved. The effects of QS signaling heterogeneity [38–41] on our results could also be explored, whether due to stochastic gene expression, low diffusivity within biofilms, or other causes. Finally, further details concerning the complex nature of QS could be incorporated into the model, including the effects of nutrient levels on QS [5].

## Materials and methods

### Model overview

We consider well-mixed populations of bacteria growing according to birth-death models with no mutation or migration (Fig 1A). Public goods are modeled implicitly through their costs and benefits as reflected in the birth rates. We write the birth rates as $\lambda_{n,m}^{\text{Pr}}$ and $\lambda_{n,m}^{\text{Ch}}$ for producers and cheaters, respectively. The subscript *n* and *m* are the number of producers and cheaters present, reflecting the dependence of the rates on the population state. The death rates are $\mu_{n,m}^{\text{Pr}}$ and $\mu_{n,m}^{\text{Ch}}$. See Eqs 1–4 for full forms of the birth and death rates.

Our model accounts for AI and public good production and degradation as well as density-dependent fitness benefits of public goods directly in the birth rates. This simplification rests on several assumptions. We assume that the timescales associated with AI and public good production and degradation are much faster than those of births and deaths. Under this assumption, the AI and public good concentrations quickly reach a steady state upon a bacterial birth or death, so that the effects of both concentrations on the birth rates is only a function of the state of the population. Similarly, the growth benefits of public goods are assumed to be a function of the current population state, namely a non-decreasing, saturating function of *n*. Another assumption is that all individuals within a given strain (producer or cheater) exhibit exactly the same birth and death rates. In reality, the stochasticity of subcellular events and diffusion of extracellular molecules leads to heterogeneous growth rates even in clonal populations [41]. Our approach simplifies analysis while providing a description of mean birth and death rates.

In order to evaluate the long-term eco-evolutionary fates of mixed producer-cheater populations, we calculate the cheater fixation probability (*π*^Ch^), mean first passage time to cheater fixation (*τ*^Ch^), and mean population extinction time (T) for all pairs of *n* producers and *m* cheaters where *n* + *m* ≤ *N*. We define *N* as a maximal population size large enough that the line *n* + *m* ≤ *N* effectively acts as a reflecting boundary. An appropriate choice of *N* then depends on the birth and death rates used. Solutions of all three quantities of interest come from solving backward Kolmogorov equations, as detailed in the Materials and Methods section. We validate the results from these solutions through stochastic simulations [42, 43].

### Birth and death rates

We use birth-death models to describe the population dynamics of a bacterial colony with both public good producers and cheaters. We assume the timescales of both autoinducer and public good production and degradation are much shorter than those of population growth. Consequently, we write per capita birth and death rates solely as a function of the number of producers and cheaters in the population. We also assume that QS is sensitive to population density. While a number of other explanations for the function of QS exist, as noted in the Introduction, all of them depend to some degree on population density.

The birth and death rates in our models take into account the costs and benefits of public good production. We use λ to denote birth rates and *μ* to denote death rates, with the superscript Pr for producers and Ch for cheaters. Let *n* be the number of producers in the population and *m* be the number of cheaters. Let λ_0_ be the birth rate due to an alternative energy source that does not require proteases for bacteria to utilize, and let *μ*_0_ be the constant death rate. We assume that the per-capita death rate increases linearly with the total population size through some density-dependent mechanism such as the production of toxic byproducts. We denote the maximal birth rate due to nutrients derived from protease activity as *g* and the additional birth rate gained by producers if they have preferential access to public goods (due to, for example, slow diffusion) as *a*. We denote the maximal cost of protease production, in terms of growth rate, as *c*. Parameters *K*_g_ and *K*_a_ control the number of producers for which protease-derived growth rate and protease production are half-maximal, respectively. Finally, *h*_g_ and *h*_a_ control the shape of the protease-derived growth rate and protease production rate functions. The full birth and death rates are

$$\begin{array}{cc} \lambda_{n,m}^{\text{Pr}} & =\text{max}([\lambda_{0}+(\left(g+a\right)\frac{{\left(n/K_{\text{g}}\right)}^{h_{\text{g}}}}{1+{\left(n/K_{\text{g}}\right)}^{h_{\text{g}}}}-c)\frac{{\left(n/K_{\text{a}}\right)}^{h_{\text{a}}}}{1+{\left(n/K_{\text{a}}\right)}^{h_{\text{a}}}}]n,0) \end{array}$$ (1)

$$\begin{array}{cc} \mu_{n,m}^{\text{Pr}} & =\mu_{0}\left(n+m\right)n \end{array}$$ (2)

$$\begin{array}{cc} \lambda_{n,m}^{\text{Ch}} & =[\lambda_{0}+g\frac{{\left(n/K_{\text{g}}\right)}^{h_{\text{g}}}}{1+{\left(n/K_{\text{g}}\right)}^{h_{\text{g}}}}\frac{{\left(n/K_{\text{a}}\right)}^{h_{\text{a}}}}{1+{\left(n/K_{\text{a}}\right)}^{h_{\text{a}}}}]m \end{array}$$ (3)

$$\begin{array}{cc} \mu_{n,m}^{\text{Ch}} & =\mu_{0}\left(n+m\right)m. \end{array}$$ (4)

The Hill function forms of these rates are similar to those in previous work [44, 45]. The strategy taken by a particular strain in controlling public good production is controlled by the parameters *K*_a_ and *h*_a_. The parameter *K*_a_ controls when public good production is half-maximal, while *h*_a_ controls the shape of the activation curve. For an “always-on” strain, we take the limit *K*_a_ → 0 so that public good production is always maximal, independent of the producer population size. For a “no production” strain (equivalent to a cheater that produces autoinducer), we take *K*_a_ → ∞ so that the public good is never produced.

We set the maximum birth rate due to public goods to *g* = 1 in all cases, so that all other birth rate parameters can be considered to be in units of maximum public good birth rate. In general we consider the parameters *g*, *K*_g_, *h*_g_, *c*, and λ_0_ to be properties of the public good and environment which cannot be changed by bacterial strategy. Similarly, we consider the parameter *μ*_0_ to be a joint property of the bacterial species and the environment. As we have stated, *K*_a_ and *h*_a_ constitute the choice of public good regulation strategy.

For the special case where cheaters produce autoinducer but not public goods, we modify the birth rates to

$$\begin{array}{cc} \lambda_{n,m}^{\text{Pr}} & =\text{max}([\lambda_{0}+(\left(g+a\right)\frac{(n/K_{\text{g}}{)}^{h_{\text{g}}}}{1+{\left(n/K_{\text{g}}\right)}^{h_{\text{g}}}}-c)\frac{{\left(\left(n+m\right)/K_{\text{a}}\right)}^{h_{\text{a}}}}{1+{\left(\left(n+m\right)/K_{\text{a}}\right)}^{h_{\text{a}}}}]n,0) \end{array}$$ (5)

$$\begin{array}{cc} \lambda_{n,m}^{\text{Ch}} & =[\lambda_{0}+g\frac{(n/K_{\text{g}}{)}^{h_{\text{g}}}}{1+{\left(n/K_{\text{g}}\right)}^{h_{\text{g}}}}\frac{{\left(\left(n+m\right)/K_{\text{a}}\right)}^{h_{\text{a}}}}{1+{\left(\left(n+m\right)/K_{\text{a}}\right)}^{h_{\text{a}}}}]m \end{array}$$ (6)

under the assumption that producers and cheaters contribute autoinducer equally. The parameter values we have used are summarized in S1 Table.

### Fixation probabilities and mean first passage times

The dynamics of the probability distribution over *n* and *m* at time *t* given *n*′ and *m*′ at an earlier time *s* (with *s* < *t*) is governed by the forward Kolmogorov equation

$$\begin{array}{cc} \frac{\partial }{\partial t}P\left(n,m,t\;|\;n^{\prime },m^{\prime },s\right)= & \;\lambda_{n-1,m}^{\text{Pr}}P\left(n-1,m,t\;|\;n^{\prime },m^{\prime },s\right)\\ & +\mu_{n+1,m}^{\text{Pr}}P\left(n+1,m,t\;|\;n^{\prime },m^{\prime },s\right)\\ & +\lambda_{n,m-1}^{\text{Ch}}P\left(n,m-1,t\;|\;n^{\prime },m^{\prime },s\right)\\ & +\mu_{n,m+1}^{\text{Ch}}P\left(n,m+1,t\;|\;n^{\prime },m^{\prime },s\right)\\ & -(\lambda_{n,m}^{\text{Pr}}+\mu_{n,m}^{\text{Pr}}+\lambda_{n,m}^{\text{Ch}}+\mu_{n,m}^{\text{Ch}})P\left(n,m,t\;|\;n^{\prime },m^{\prime },s\right) \end{array}$$ (7)

where *n*, *m*, *n*′, *m*′ ∈ {0, 1, 2, …}. For birth and death rates linear in *n* and *m*, the system of coupled differential equations represented by Eq 7 can be solved exactly using a generating function approach [46]. However, the birth and death rates required for our purposes are nonlinear, and we adopt a computational approach.

The main quantities of interest in this work are the probability of cheater fixation and the mean population extinction time. These quantities can be calculated from the backward Kolmogorov equation

$$\begin{array}{cc} \frac{\partial }{\partial s}P\left(n,m,t\;|\;n^{\prime },m^{\prime },s\right)= & \;\lambda_{n^{\prime },m^{\prime }}^{\text{Pr}}(P\left(n,m,t\;|\;n^{\prime }+1,m^{\prime },s\right)-P\left(n,m,t\;|\;n^{\prime },m^{\prime },s\right))\\ & +\mu_{n^{\prime },m^{\prime }}^{\text{Pr}}(P\left(n,m,t\;|\;n^{\prime }-1,m^{\prime },s\right)-P\left(n,m,t\;|\;n^{\prime },m^{\prime },s\right))\\ & +\lambda_{n^{\prime },m^{\prime }}^{\text{Ch}}(P\left(n,m,t\;|\;n^{\prime },m^{\prime }+1,s\right)-P\left(n,m,t\;|\;n^{\prime },m^{\prime },s\right))\\ & +\mu_{n^{\prime },m^{\prime }}^{\text{Ch}}(P\left(n,m,t\;|\;n^{\prime },m^{\prime }-1,s\right)-P\left(n,m,t\;|\;n^{\prime },m^{\prime },s\right)). \end{array}$$ (8)

We define a maximum pure producer or cheater population size *N* and construct an (*N* + 1)^2^ × (*N* + 1)^2^ matrix describing the transition rates between all states and use it to solve for our quantities of interest. Given the birth and death rates we use, we assume that the approximation introduced by considering a finite rate matrix is reasonable when net growth rates are negative and have a large magnitude in all states where *m* = *N* or *n* = *N*. For the probability that cheaters will fix in the population, we solve [46]

$$\begin{array}{cc} & \lambda_{n,m}^{\text{Pr}}(\pi_{n+1,m}^{\text{Ch}}-\pi_{n,m}^{\text{Ch}})+\mu_{n,m}^{\text{Pr}}(\pi_{n-1,m}^{\text{Ch}}-\pi_{n,m}^{\text{Ch}})\\ & +\lambda_{n,m}^{\text{Ch}}(\pi_{n,m+1}^{\text{Ch}}-\pi_{n,m}^{\text{Ch}})+\mu_{n,m}^{\text{Ch}}(\pi_{n,m-1}^{\text{Ch}}-\pi_{n,m}^{\text{Ch}})=0 \end{array}$$ (9)

with boundary conditions

$$\begin{array}{cc} \pi_{0,m}^{\text{Ch}} & =1,\;\text{for}\;n=0\;\text{and}\;0<m\leq N \end{array}$$ (10)

$$\begin{array}{cc} \pi_{n,0}^{\text{Ch}} & =0,\;\text{for}\;0\leq n\leq N\;\text{and}\;m=0. \end{array}$$ (11)

For the mean first passage time conditioned on cheater fixation, we solve

$$\begin{array}{cc} & \lambda_{n,m}^{\text{Pr}}(\theta_{n+1,m}^{\text{Ch}}-\theta_{n,m}^{\text{Ch}})+\mu_{n,m}^{\text{Pr}}(\theta_{n-1,m}^{\text{Ch}}-\theta_{n,m}^{\text{Ch}})\\ & +\lambda_{n,m}^{\text{Ch}}(\theta_{n,m+1}^{\text{Ch}}-\theta_{n,m}^{\text{Ch}})+\mu_{n,m}^{\text{Ch}}(\theta_{n,m-1}^{\text{Ch}}-\theta_{n,m}^{\text{Ch}})=-\pi_{n,m}^{\text{Ch}} \end{array}$$ (12)

with the condition

$$\begin{array}{c} \theta_{n,m}^{\text{Ch}}=0,\;\text{for}\;n=0\;\text{or}\;m=0, \end{array}$$ (13)

and then find the conditional mean first passage time to cheater fixation according to

$$\begin{array}{c} \tau_{n,m}^{\text{Ch}}=\frac{\theta_{n,m}^{\text{Ch}}}{\pi_{n,m}^{\text{Ch}}}. \end{array}$$ (14)

Similarly, for the mean extinction time we solve

$$\begin{array}{cc} & \lambda_{n,m}^{\text{Pr}}(T_{n+1,m}-T_{n,m})+\mu_{n,m}^{\text{Pr}}(T_{n-1,m}-T_{n,m})\\ & +\lambda_{n,m}^{\text{Ch}}(T_{n,m+1}-T_{n,m})+\mu_{n,m}^{\text{Ch}}(T_{n,m-1}-T_{n,m})=-1 \end{array}$$ (15)

with the condition

$$\begin{array}{c} T_{n,m}=0,\;\text{for}\;n=0\;\text{and}\;m=0. \end{array}$$ (16)

Directly solving the linear system of equations represented by Eq 15 can lead to numerical instability when the mean extinction times are large. To account for these numerical issues we also calculated the mean extinction time using the analytical formula for pure producer or cheater populations with adjoint reflecting boundary conditions at *n* = *N* and *m* = *N* [47–49]

$$\begin{array}{cc} T_{n,0} & =\sum_{i=1}^{n}(\frac{1}{\mu_{i,0}^{\text{Pr}}}+\sum_{j=1}^{N-i}\frac{1}{\mu_{i+j,0}^{\text{Pr}}}\prod_{l=1}^{j}\frac{\lambda_{i+l-1,0}^{\text{Pr}}}{\mu_{i+l-1,0}^{\text{Pr}}}) \end{array}$$ (17)

$$\begin{array}{cc} T_{0,m} & =\sum_{i=1}^{m}(\frac{1}{\mu_{0,i}^{\text{Ch}}}+\sum_{j=1}^{N-i}\frac{1}{\mu_{0,i+j}^{\text{Ch}}}\prod_{l=1}^{j}\frac{\lambda_{0,i+l-1}^{\text{Ch}}}{\mu_{0,i+l-1}^{\text{Ch}}}) \end{array}$$ (18)

and direct calculations of fixation probabilities for each point along the pure producer and cheater axes. We use the notation $\pi_{n,m}^{i,0}$ and $\pi_{n,m}^{0,j}$ to indicate the probability that a population starting with *n* producers and *m* cheaters will have producers fix with *i* total producers and will have cheaters fix with *j* total cheaters, respectively. We then estimate the mean extinction time according to

$$\begin{array}{c} T_{n,m}=\sum_{i=1}^{N}\pi_{n,m}^{i,0}T_{i,0}+\sum_{j=1}^{N}\pi_{n,m}^{0,j}T_{0,j}, \end{array}$$ (19)

under the assumption that producers or cheaters will quickly fix followed by a much longer period with a pure population before extinction.

### Stochastic simulations

Stochastic simulations were performed using Gillespie’s algorithm [42, 43] implemented in the Gillespy2 Python package [50]. Each simulation was run until population extinction and was repeated 100 times in order to estimate fixation probabilities and mean first passage times.

### General methods

The figures in this article were created using Inkscape 0.92 and Matplotlib 3.1.1 [51] in a Python 3.7 Jupyter Notebook [52]. All fixation probabilities and mean first passage times were calculated using the linear system solver in the Python NumPy package [53] and the sparse matrix module of the SciPy package [54].

## Supporting information

S1 Fig The cheater fixation probabilities from Fig 2 agree with simulation results. The first row shows the cheater fixation probabilities for A) quorum sensing (QS) and B) always on (AO) strategies, directly reproduced from Fig 2. The second row shows cheater fixation probabilities calculated as a mean from 100 independent simulations for C) QS and D) AO strategies. Points above *n* + *m* = 70 (above the zero-net growth contour) were not calculated. See S2 Table for parameter values.
(TIFF)

S2 Fig The cheater mean first passage time to fixation from Fig 2 agree with simulation results. The first row shows the cheater fixation mean first passage times for A) quorum sensing (QS) and B) always on (AO) strategies, directly reproduced from Fig 2. The second row shows cheater fixation mean first passage times calculated as a mean from 100 independent simulations for C) QS and D) AO strategies. Points above *n* + *m* = 70 (above the zero-net growth contour) were not calculated. See S2 Table for parameter values.
(TIFF)

S3 Fig The qualitative results from Fig 2 are preserved with increased carrying capacity, with no constitutive growth (λ_0_). The first row depicts cheater fixation probability from an initial population structure of *n* producers and *m* cheaters for A) quorum sensing (QS) and B) always on (AO) strategies. The second row depicts cheater fixation probabilities calculated as a mean of 100 independent simulations for C) QS and D) AO strategies. Points above *n* + *m* = 140 (above the zero-net growth contour) were not calculated. The third row depicts mean extinction time from initial population structure for E) QS and F) AO strategies, calculated according to Eq 19. As with the results in Fig 2, QS decreases cheater fixation probability but also decreases mean extinction time as compared with AO. See S2 Table for parameter values.
(TIFF)

S4 Fig The cheater fixation probabilities from Fig 3 agree with simulation results. The first row shows the cheater fixation probabilities for A) quorum sensing (QS) and B) always on (AO) strategies, directly reproduced from Fig 3. The second row shows cheater fixation probabilities calculated as a mean from 100 independent simulations for C) QS and D) AO strategies. Points above *n* + *m* = 70 (above the zero-net growth contour) were not calculated. See S2 Table for parameter values.
(TIFF)

S5 Fig The cheater mean first passage time to fixation from Fig 3 agree with simulation results. The first row shows the cheater fixation mean first passage times for A) quorum sensing (QS) and B) always on (AO) strategies, directly reproduced from Fig 3. The second row shows cheater fixation mean first passage times calculated as a mean from 100 independent simulations for C) QS and D) AO strategies. Points above *n* + *m* = 70 (above the zero-net growth contour) were not calculated. See S2 Table for parameter values.
(TIFF)

S6 Fig The qualitative results from Fig 3 are preserved with increased carrying capacity, with constitutive growth (λ_0_ = 0.2). The first row depicts cheater fixation probability from an initial population structure of *n* producers and *m* cheaters for A) quorum sensing (QS) and B) always on (AO) strategies. The second row depicts cheater fixation probabilities calculated as a mean of 100 independent simulations for C) QS and D) AO strategies. Points above *n* + *m* = 140 (above the zero-net growth contour) were not calculated. The third row depicts mean extinction time from initial population structure for E) QS and F) AO strategies, calculated according to Eq 19. As with the results in Fig 3, QS decreases cheater fixation probability while also increasing mean extinction time as compared with AO. See S2 Table for parameter values.
(TIFF)

S7 Fig Producer advantage reduces the benefit of QS over AO with the parameters from Fig 2. A)-C) The cheater fixation probability with QS divided by the cheater fixation probability with AO. D)-F) Logarithm of the ratio of mean extinction time for QS with AO. The cheater fixation probability of QS relative to AO increases with *a* while the log-ratio of QS mean extinction time to AO mean extinction time decreases with *a*.
(TIFF)

S8 Fig Producer advantage reduces the benefit of QS over AO with the parameters from Fig 3. A)-C) The cheater fixation probability with QS divided by the cheater fixation probability with AO. D)-F) Logarithm of the ratio of mean extinction time for QS with AO. The cheater fixation probability of QS relative to AO increases with *a*, though less noticeably than in S7 Fig. Because QS is constructive in this case, the The log-ratio of QS mean extinction time to AO mean extinction time is positive, but decreases with *a*.
(TIFF)

S9 FigPhase diagram describing the fitness gains of QS over AO as a function of constitutive growth rate (λ_0_) and public good production cost (*c*).For each pair (λ_0_, *c*) we calculated the cheater fixation probability for all (*n*, *m*) pairs satisfying *n* ≥ 0, *m* ≥ 0, and *n* + *m* ≤ 100 with the QS and AO strategies. The reported number is the fraction of these (*n*, *m*) pairs where the cheater fixation probability for QS is less than for AO ($\pi_{n,m}^{QS}<\pi_{n,m}^{AO}$). For all (λ_0_, *c*) pairs except for when *c* = 0, cheater fixation probability is reduced by QS for all initial population compositions. When *c* = 0, there are a small number of initial compositions where $\pi_{n,m}^{QS}\geq \pi_{n,m}^{AO}$ which decreases as λ_0_ increases. See S2 Table for parameter values.(TIFF)

S10 FigDetailed analysis of the phase diagram from Fig 1 with *c* = 0.2 fixed and with different λ_0_ values.Top row: the red dots shown over the phase diagram indicate the (*c*, λ_0_) pairs that we examine in detail. Middle row: the ratio of QS mean extinction time to AO mean extinction time $\left(T_{QS}/T_{AO}\right)$ with the indicated values of λ_0_. The fraction of (*n*, *m*) pairs where $\left(T_{QS}>T_{AO}\right)$ is indicated as $f(T_{QS}>T_{AO})$. Bottom row: the zero expected net growth contour contours for QS producers (red), QS cheaters (orange), AO producers (black), and AO cheaters (grey). For λ_0_ = 0 the two QS zero expected net growth contour contours are identical and the two AO zero expected net growth contour contours are also identical. As λ_0_ is increased, the AO producer contour approaches the producer axis faster than the QS producer contour. See S2 Table for parameter values.(TIFF)

S11 FigDetailed analysis of the phase diagram from Fig 1 with λ_0_ = 0.2 fixed and with different *c* values.Top row: the red dots shown over the phase diagram indicate the (*c*, λ_0_) pairs that we examine in detail. Middle row: the ratio of QS mean extinction time to AO mean extinction time $\left(T_{QS}/T_{AO}\right)$ with the indicated values of *c*. The fraction of (*n*, *m*) pairs where $\left(T_{QS}>T_{AO}\right)$ is indicated as $f(T_{QS}>T_{AO})$. Bottom row: the zero expected net growth contour contours for QS producers (red), QS cheaters (orange), AO producers (black), and AO cheaters (grey). At λ_0_ = 0.075 the diagonal zero expected net growth contour contour near the origin appears for QS, and moves further from the origin as λ_0_ increases from there. See S2 Table for parameter values.(TIFF)

S12 FigAnalysis of the phase diagram in Fig 1B, with varied criteria for the relative values of $T_{\text{QS}}$ and $T_{\text{AO}}$.We define a tolerance, *δ*, so that the above plots display the fraction of (*n*, *m*) pairs with $\text{log}(T_{n,m:\text{QS}}/T_{n,m:\text{AO}})>\delta$. Fig 1B corresponds to *δ* = 0. With slightly larger *δ*, the region with higher fraction around λ_0_ = 0.05, *c* > 0.2 rapidly disappears. This demonstrates that the QS mean extinction times are only slightly larger in this region.(TIFF)

S13 FigThe log ratio of the QS mean extinction time to AO mean extinction time with several initial population compositions, reflecting intermediate calculations towards producing Fig 1B and S12 Fig.At low *c* values, $T_{n,m:\text{QS}}<T_{n,m:\text{AO}}$ for all initial conditions, reflecting the destructive region of Fig 1B. With large *c* and λ_0_, $T_{n,m:\text{QS}}>T_{n,m:\text{AO}}$ reflecting the constructive region. All other combinations of *c* and λ_0_ lead to relatively minor differences between $T_{n,m:\text{QS}}$ and $T_{n,m:\text{AO}}$.(TIFF)

S14 FigMean extinction time $T_{1,0:\text{QS}}$ in a colonization scenario as a function of *K*_*a*_, with an initial *n* = 1 producer and *m* = 0 cheaters.The quantity $T_{1,0:\text{QS}}$ represents the ability of a single producer to succeed in establishing a persistent colony in the absence of cheaters. The *K*_*a*_ value that maximizes mean extinction time is weakly dependent on λ_0_ and increases with larger *c*, reflecting the larger net benefit of delaying public good activation with higher costs until the public good is more beneficial (determined by the *K*_*g*_ and *h*_*g*_ parameters). At *c* = 0, the AO strategy maximizes the mean extinction time, as there is no cost whatsoever to public good production. Dashed vertical line: optimal value of *K*_*a*_ when λ_0_ = 0.25.(TIFF)

S15 FigCheater fixation probability in an invasion scenario as a function of *K*_*a*_, with an initial $n^{⋆}=\text{round}(\frac{\lambda_{0}+g-c}{\mu })$ producers (approximate equilibrium value in the absence of cheaters) and *m* = 1 cheater.With no cost of public good production (*c* = 0), *K*_*a*_ has no effect on cheater fixation probability. For all other examined costs, the fixation probability monotonically decreases as a function of *K*_*a*_ for all examined λ_0_ values. This behavior suggests that the NP strategy, characterized by *K*_*a*_ → ∞, minimizes the cheater fixation probability.(TIFF)

S16 FigTrade-offs between suppressing cheaters and decreasing the time to stochastic clearance of a pure producer population, as controlled by *K*_*a*_, are present for λ_0_ > 0 but absent when λ_0_ = 0.In the left figure, increasing *K*_*a*_ has no effect on mean extinction time, so that loss of public good production altogether (*K*_*a*_ → ∞) minimizes cheater fixation probability compared with AO. Here, we calculated the log-ratio of mean extinction times, $\text{log}(T_{1,0:\text{QS}}/T_{1,0:\text{AO}})$, and the log-ratio of cheater fixation probabilities, $\text{log}(\pi_{n^{⋆},1:\text{QS}}^{\text{Ch}}/\pi_{n^{⋆},1:\text{AO}}^{\text{Ch}})$, for all 625 different combinations of *K*_*g*_, *K*_*a*_ ∈ {10, 15, 20, 25, 30} and *h*_*a*_, *h*_*g*_ ∈ {1, 2, 3, 4, 5}. Each point on the plot represents the results for one of the 625 parameter combinations. We calculated mean extinction times for *n* = 1 producers and *m* = 0 cheaters, corresponding to the case where a single producer is colonizing an otherwise empty region of space. We calculated cheater fixation probabilities for $n^{⋆}=\text{round}(\frac{\lambda_{0}+g-c}{\mu })$ producers and *m* = 1 cheater, the relevant initial population composition for the case of a single cheater arising by mutation in a population or producers. The highlighted points show how varying *K*_*a*_ alone affects the results (*K*_*a*_ = 15, used in Figs 2 and 3, is indicated by a star), with *K*_*g*_, *h*_*g*_, and *h*_*a*_ fixed as in Figs 2 and 3.(TIFF)

S1 Table Model parameters used in main text figures. (XLSX)

S2 Table Model parameters used in supplemental figures. (XLSX)
