# Supplementary figures and images for: Cheater suppression and stochastic clearance through quorum sensing
Source: PLoS Comput Biol. 2022 Jul 28;18(7):e1010292. doi: 10.1371/journal.pcbi.1010292 (PMC9333318; doi:10.1371/journal.pcbi.1010292)

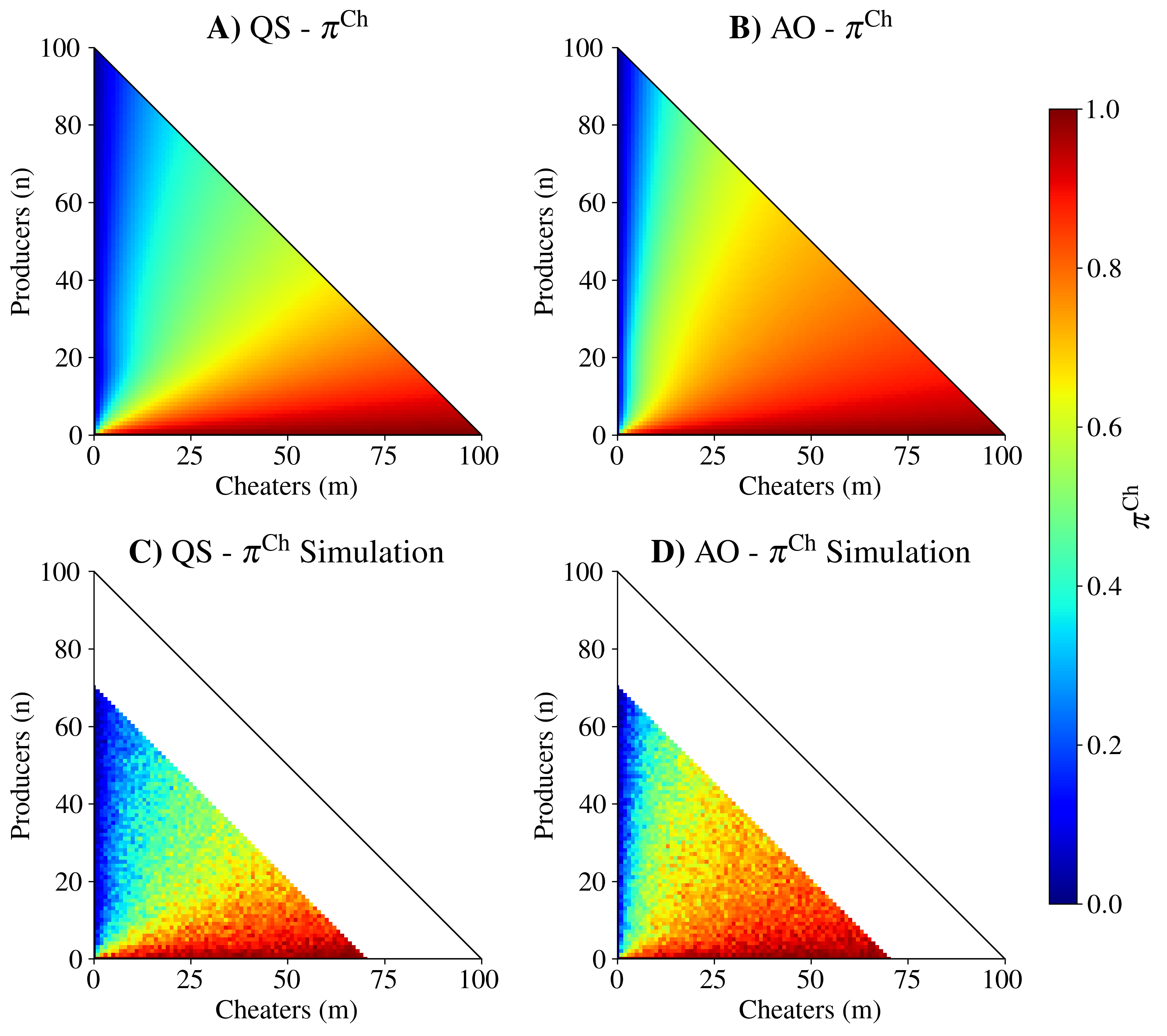

Supplement: S1 Fig — The first row shows the cheater fixation probabilities for A) quorum sensing (QS) and B) always on (AO) strategies, directly reproduced from Fig 2. The second row shows cheater fixation probabilities calculated as a mean from 100 independent simulations for C) QS and D) AO strategies. Points above n + m = 70 (above the zero-net growth contour) were not calculated. See S2 Table for parameter values. (TIFF) [file pcbi.1010292.s001.tiff]

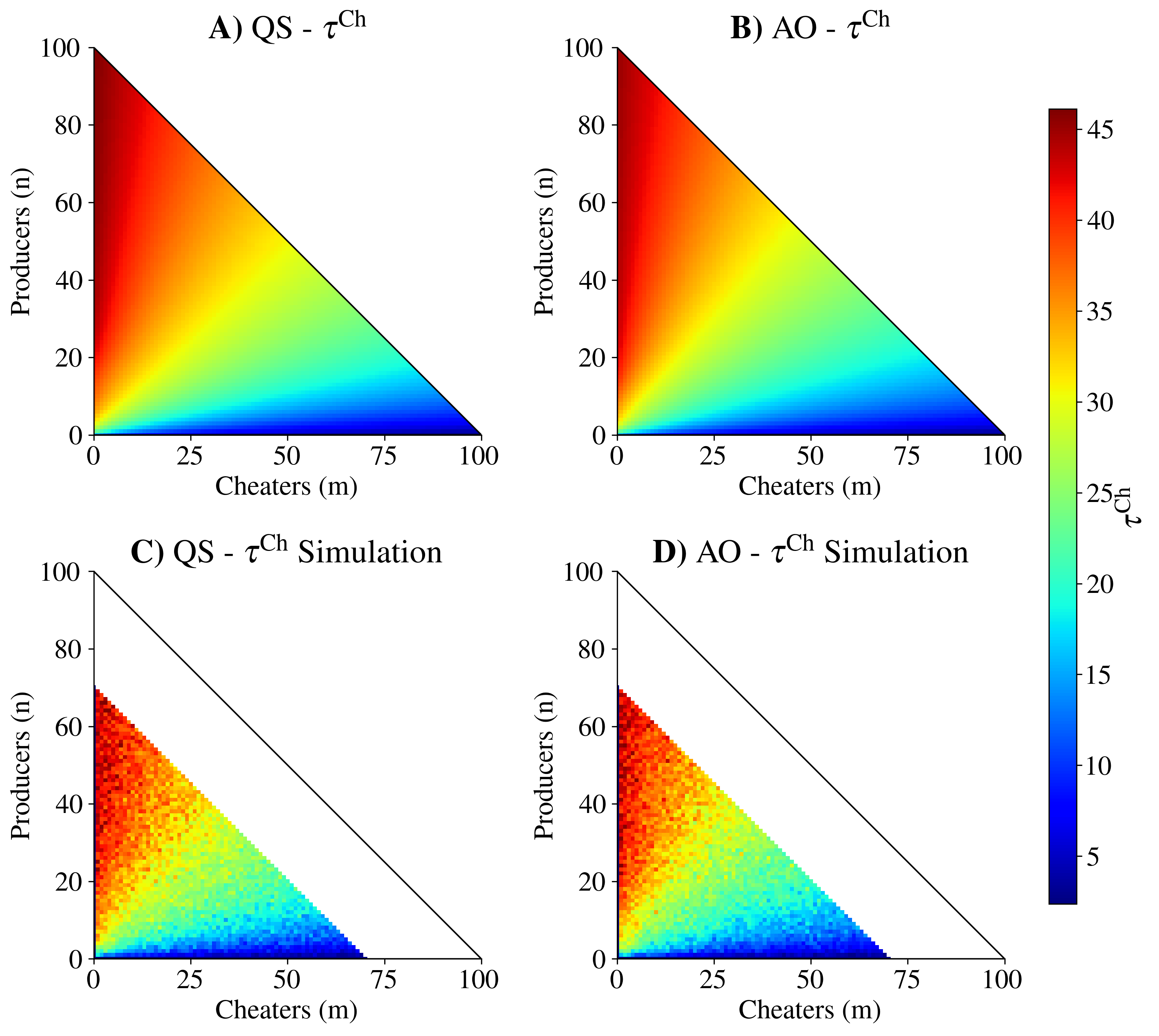

Supplement: S2 Fig — The first row shows the cheater fixation mean first passage times for A) quorum sensing (QS) and B) always on (AO) strategies, directly reproduced from Fig 2. The second row shows cheater fixation mean first passage times calculated as a mean from 100 independent simulations for C) QS and D) AO strategies. Points above n + m = 70 (above the zero-net growth contour) were not calculated. See S2 Table for parameter values. (TIFF) [file pcbi.1010292.s002.tiff]

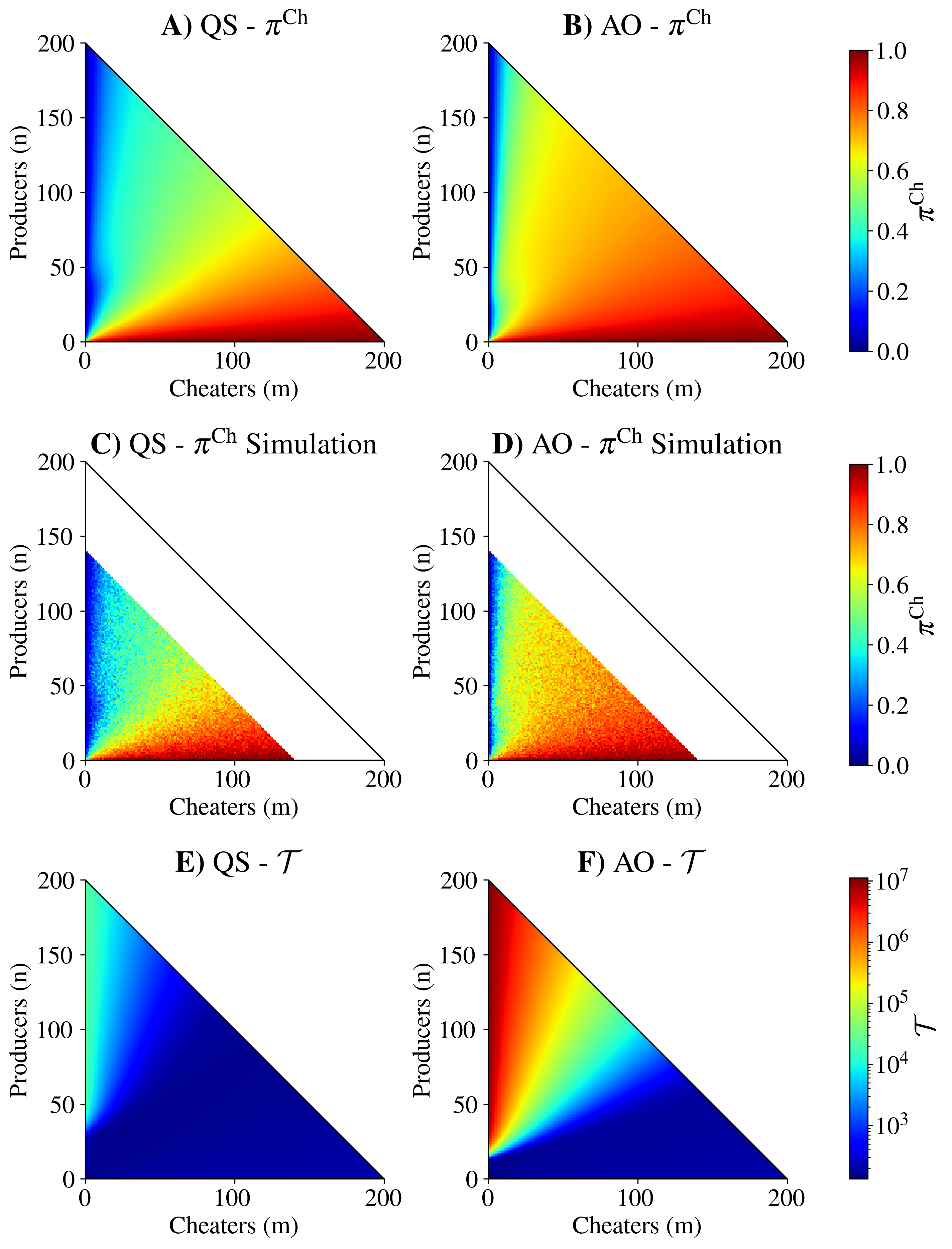

Supplement: S3 Fig — The first row depicts cheater fixation probability from an initial population structure of n producers and m cheaters for A) quorum sensing (QS) and B) always on (AO) strategies. The second row depicts cheater fixation probabilities calculated as a mean of 100 independent simulations for C) QS and D) AO strategies. Points above n + m = 140 (above the zero-net growth contour) were not calculated. The third row depicts mean extinction time from initial population structure for E) QS and F) AO strategies, calculated according to Eq 19. As with the results in Fig 2, QS decreases cheater fixation probability but also decreases mean extinction time as compared with AO. See S2 Table for parameter values. (TIFF) [file pcbi.1010292.s003.tiff]

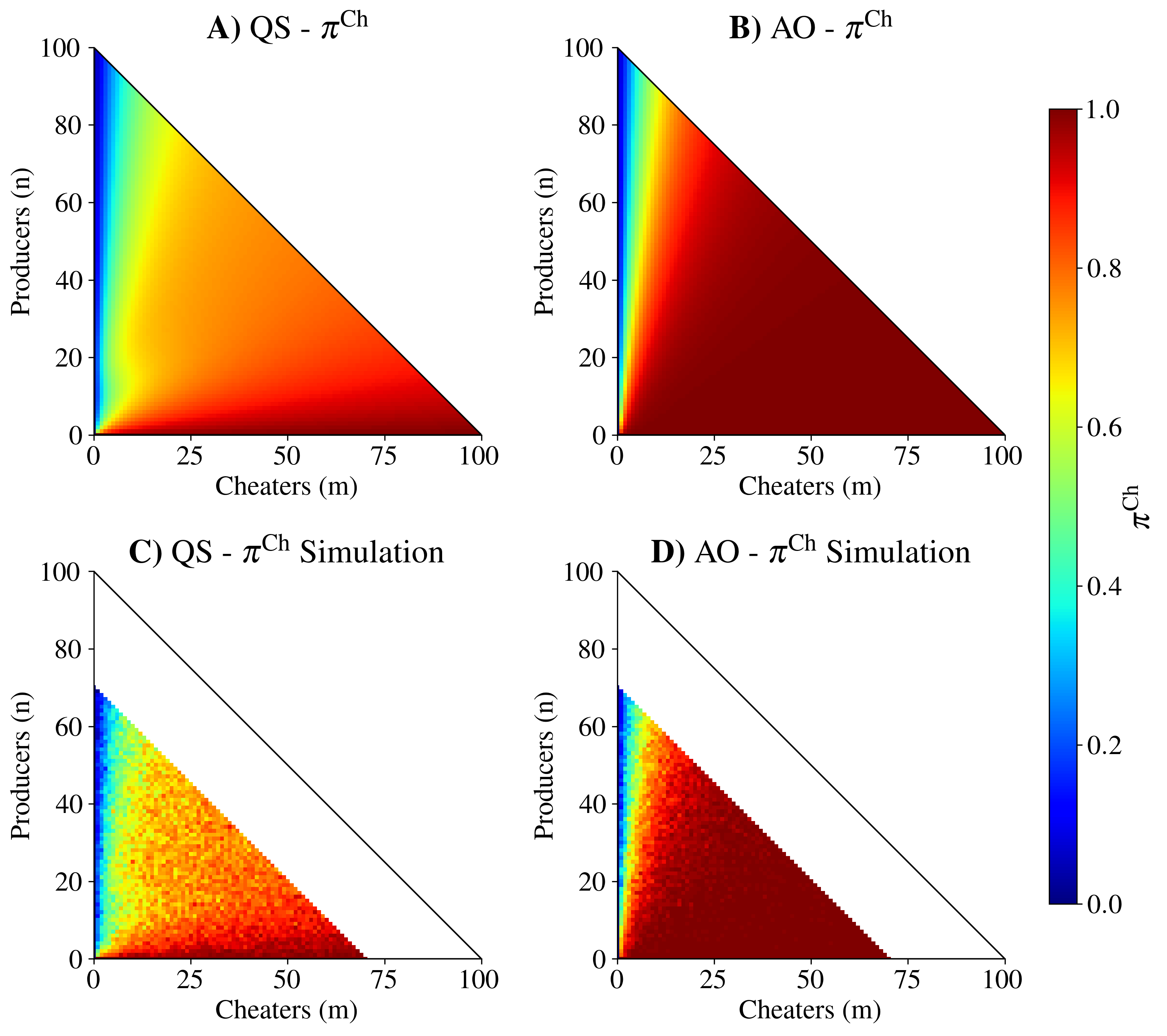

Supplement: S4 Fig — The first row shows the cheater fixation probabilities for A) quorum sensing (QS) and B) always on (AO) strategies, directly reproduced from Fig 3. The second row shows cheater fixation probabilities calculated as a mean from 100 independent simulations for C) QS and D) AO strategies. Points above n + m = 70 (above the zero-net growth contour) were not calculated. See S2 Table for parameter values. (TIFF) [file pcbi.1010292.s004.tiff]

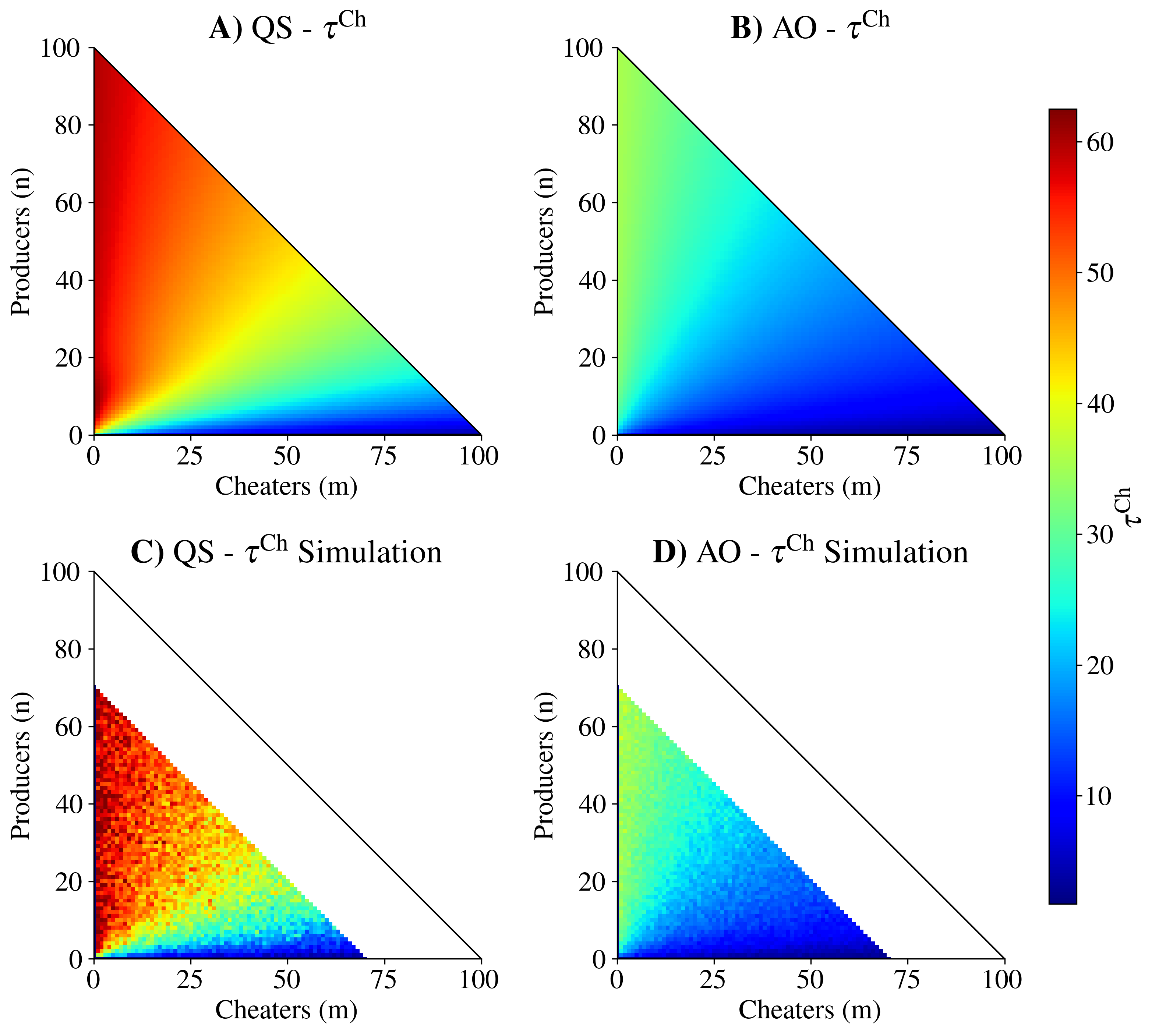

Supplement: S5 Fig — The first row shows the cheater fixation mean first passage times for A) quorum sensing (QS) and B) always on (AO) strategies, directly reproduced from Fig 3. The second row shows cheater fixation mean first passage times calculated as a mean from 100 independent simulations for C) QS and D) AO strategies. Points above n + m = 70 (above the zero-net growth contour) were not calculated. See S2 Table for parameter values. (TIFF) [file pcbi.1010292.s005.tiff]

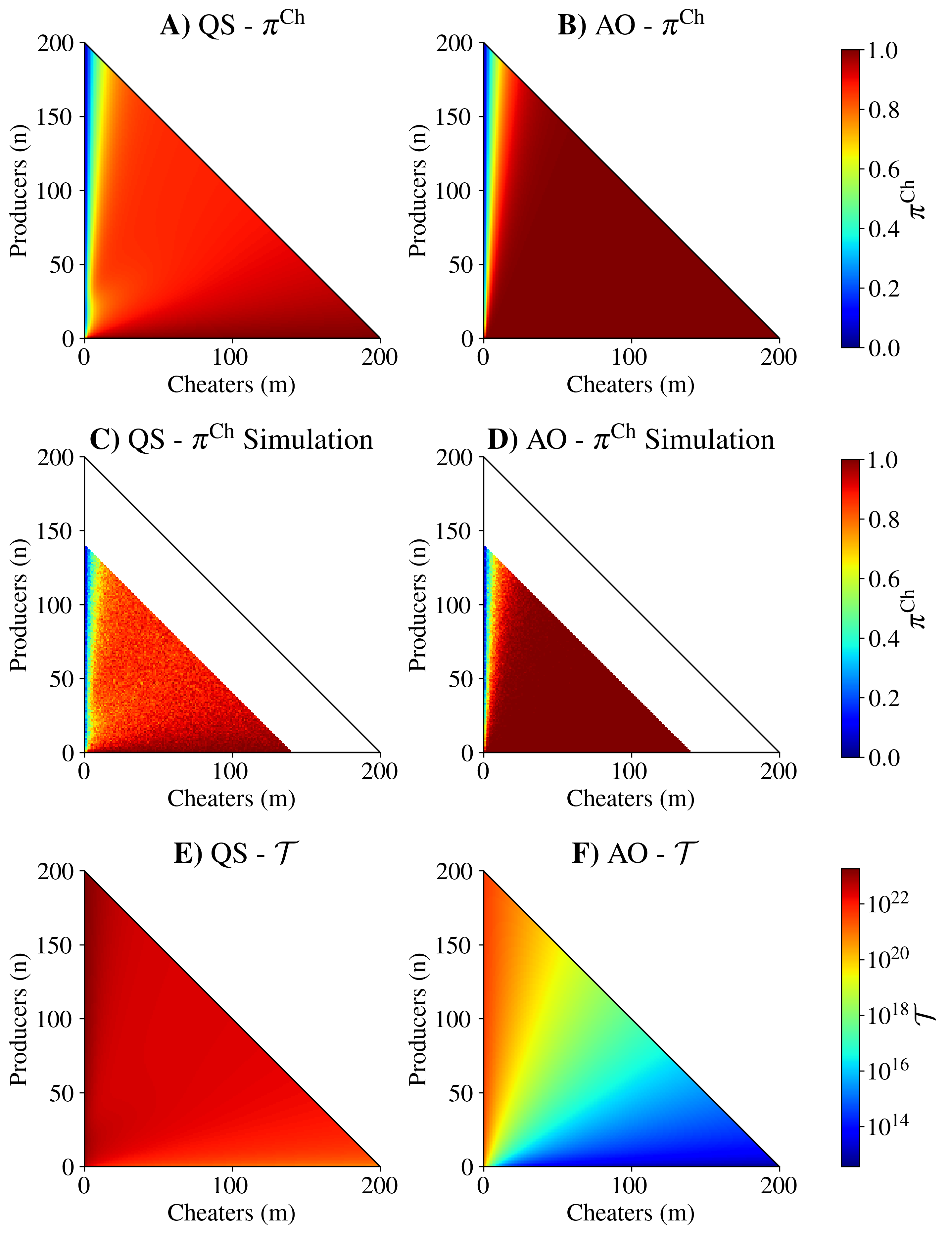

Supplement: S6 Fig — The first row depicts cheater fixation probability from an initial population structure of n producers and m cheaters for A) quorum sensing (QS) and B) always on (AO) strategies. The second row depicts cheater fixation probabilities calculated as a mean of 100 independent simulations for C) QS and D) AO strategies. Points above n + m = 140 (above the zero-net growth contour) were not calculated. The third row depicts mean extinction time from initial population structure for E) QS and F) AO strategies, calculated according to Eq 19. As with the results in Fig 3, QS decreases cheater fixation probability while also increasing mean extinction time as compared with AO. See S2 Table for parameter values. (TIFF) [file pcbi.1010292.s006.tiff]

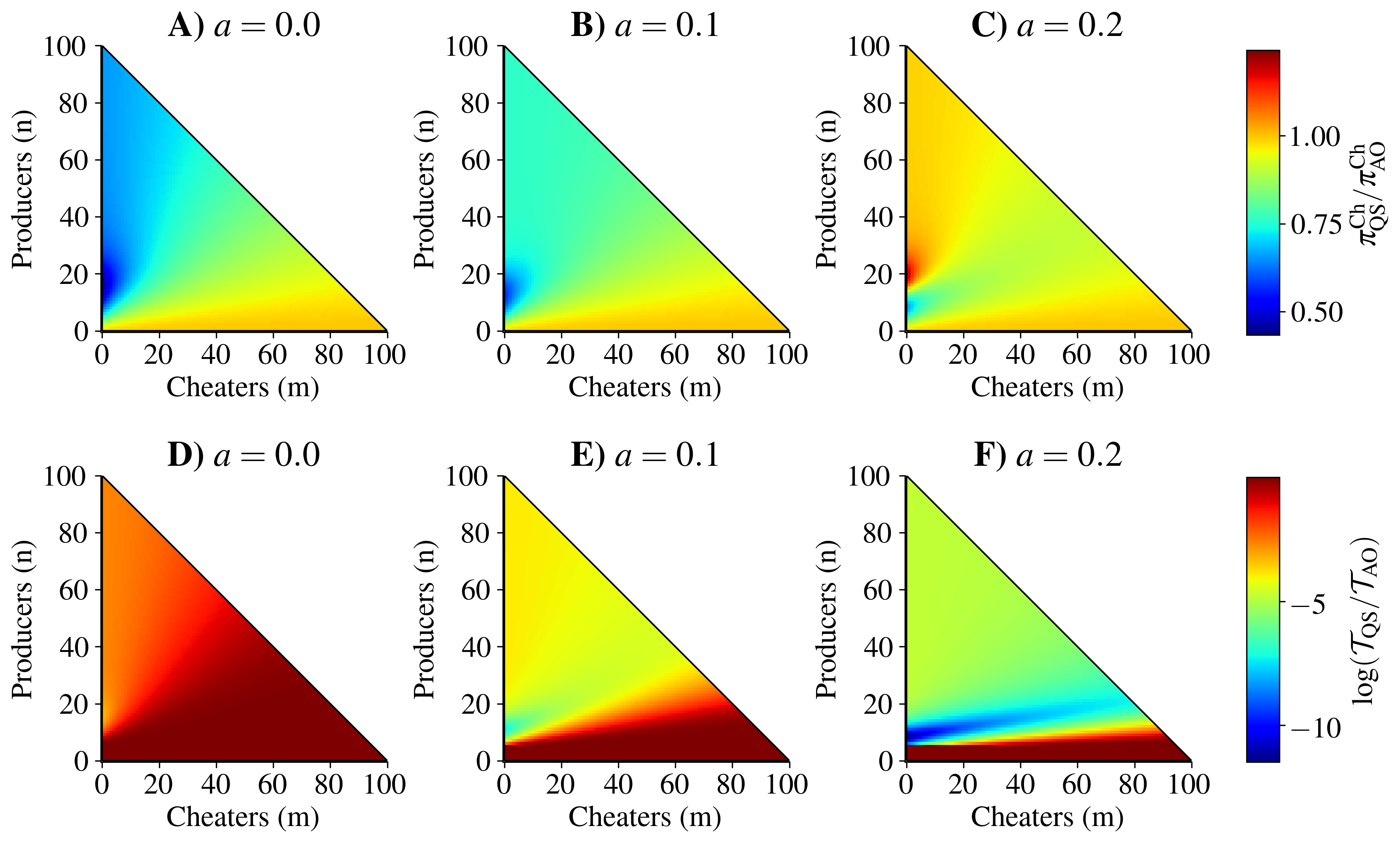

Supplement: S7 Fig — A)-C) The cheater fixation probability with QS divided by the cheater fixation probability with AO. D)-F) Logarithm of the ratio of mean extinction time for QS with AO. The cheater fixation probability of QS relative to AO increases with a while the log-ratio of QS mean extinction time to AO mean extinction time decreases with a. (TIFF) [file pcbi.1010292.s007.tiff]

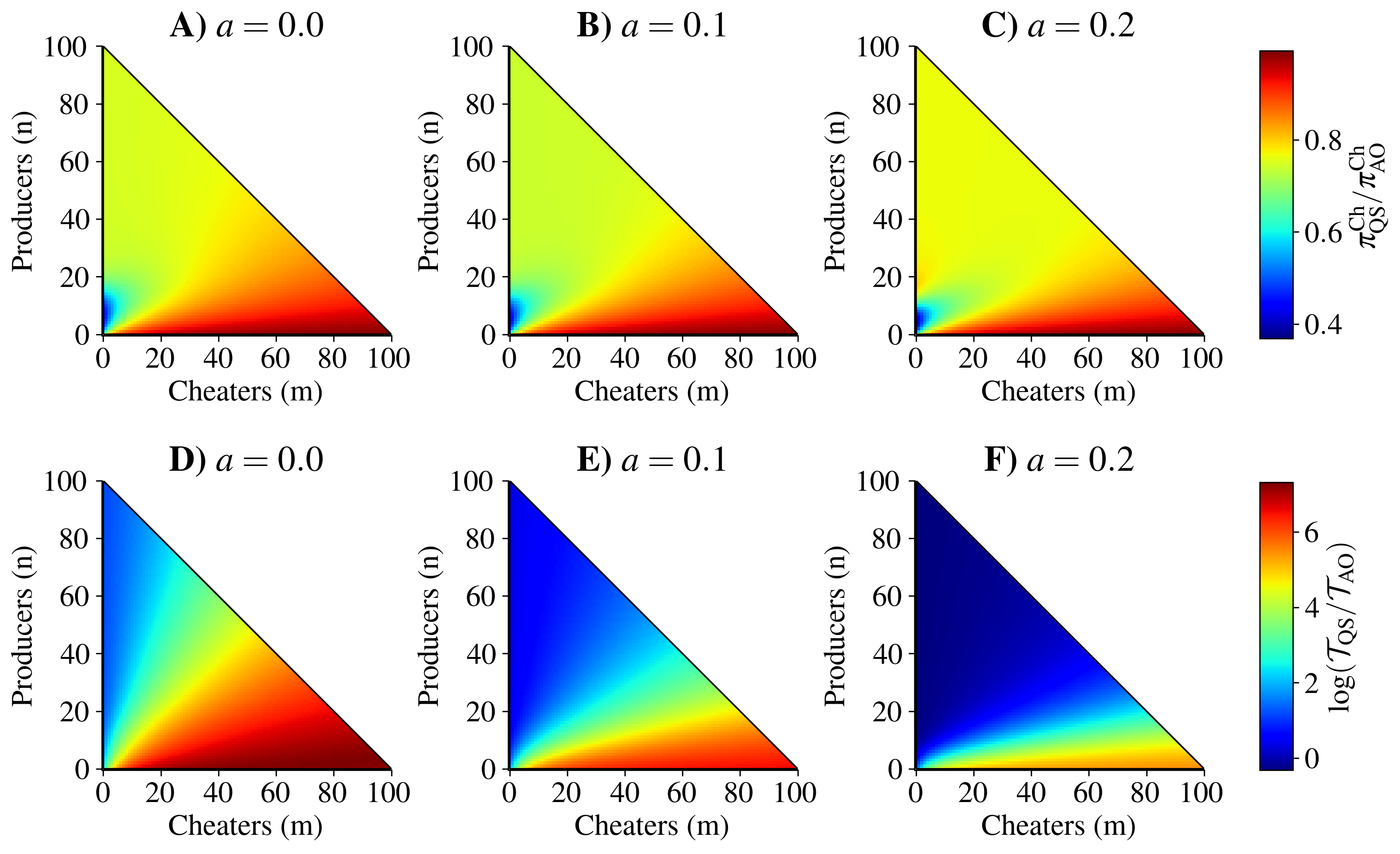

Supplement: S8 Fig — A)-C) The cheater fixation probability with QS divided by the cheater fixation probability with AO. D)-F) Logarithm of the ratio of mean extinction time for QS with AO. The cheater fixation probability of QS relative to AO increases with a, though less noticeably than in S7 Fig. Because QS is constructive in this case, the The log-ratio of QS mean extinction time to AO mean extinction time is positive, but decreases with a. (TIFF) [file pcbi.1010292.s008.tiff]

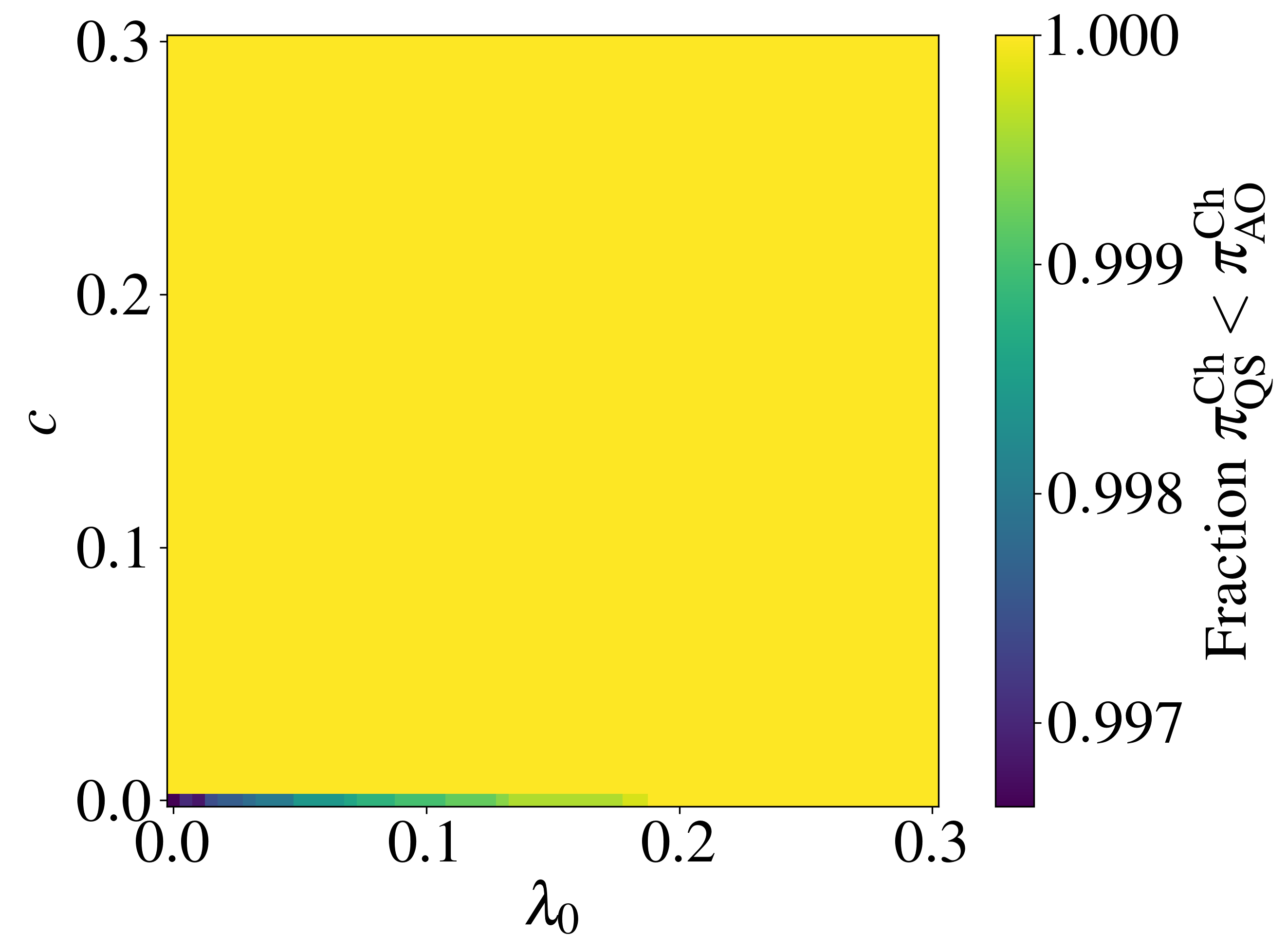

Supplement: S9 Fig — For each pair (λ0, c) we calculated the cheater fixation probability for all (n, m) pairs satisfying n ≥ 0, m ≥ 0, and n + m ≤ 100 with the QS and AO strategies. The reported number is the fraction of these (n, m) pairs where the cheater fixation probability for QS is less than for AO (πn,mQS<πn,mAO). For all (λ0, c) pairs except for when c = 0, cheater fixation probability is reduced by QS for all initial population compositions. When c = 0, there are a small number of initial compositions where πn,mQS≥πn,mAO which decreases as λ0 increases. See S2 Table for parameter values. (TIFF) [file pcbi.1010292.s009.tiff]

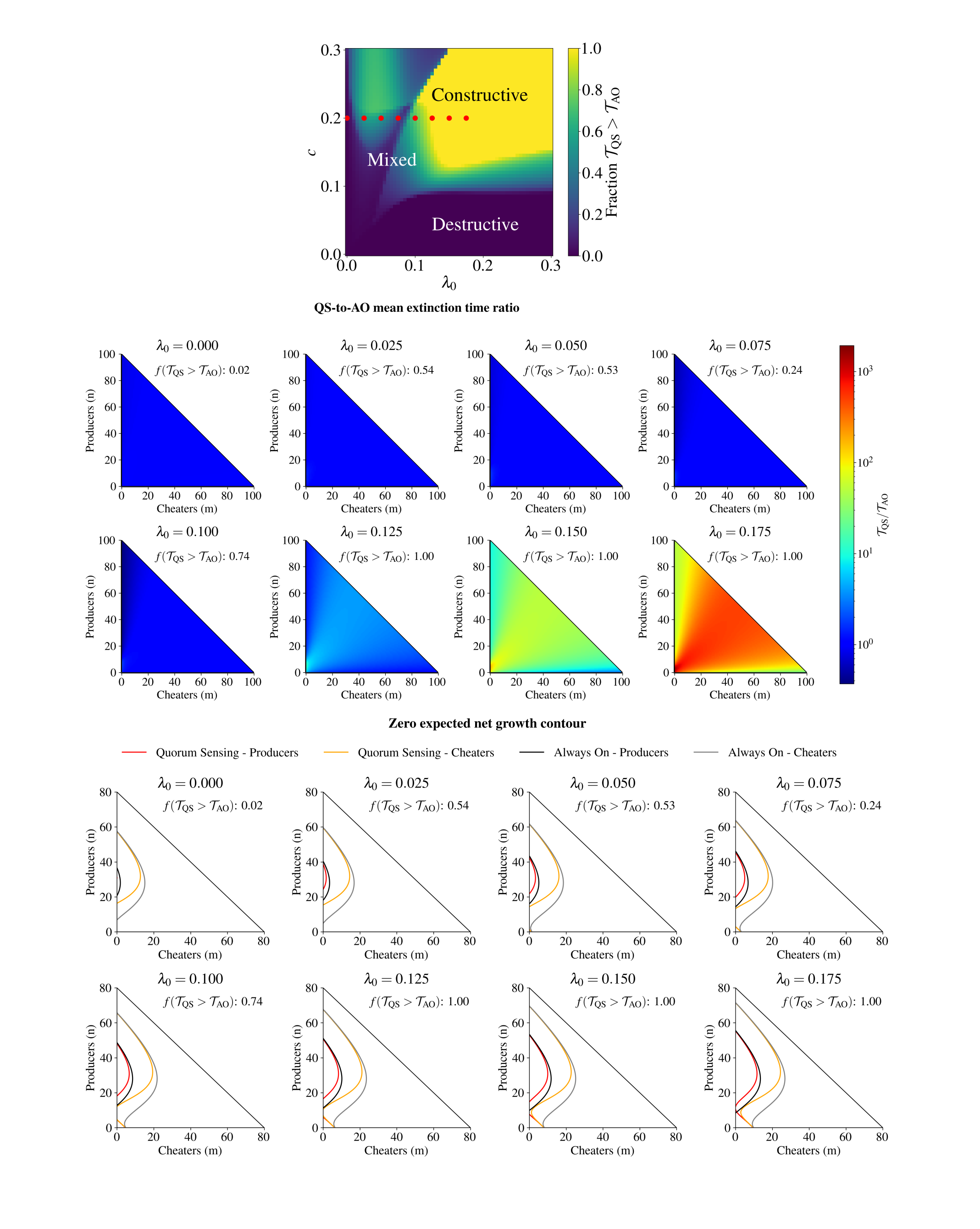

Supplement: S10 Fig — Top row: the red dots shown over the phase diagram indicate the (c, λ0) pairs that we examine in detail. Middle row: the ratio of QS mean extinction time to AO mean extinction time (TQS/TAO) with the indicated values of λ0. The fraction of (n, m) pairs where (TQS>TAO) is indicated as f(TQS>TAO). Bottom row: the zero expected net growth contour contours for QS producers (red), QS cheaters (orange), AO producers (black), and AO cheaters (grey). For λ0 = 0 the two QS zero expected net growth contour contours are identical and the two AO zero expected net growth contour contours are also identical. As λ0 is increased, the AO producer contour approaches the producer axis faster than the QS producer contour. See S2 Table for parameter values. (TIFF) [file pcbi.1010292.s010.tiff]

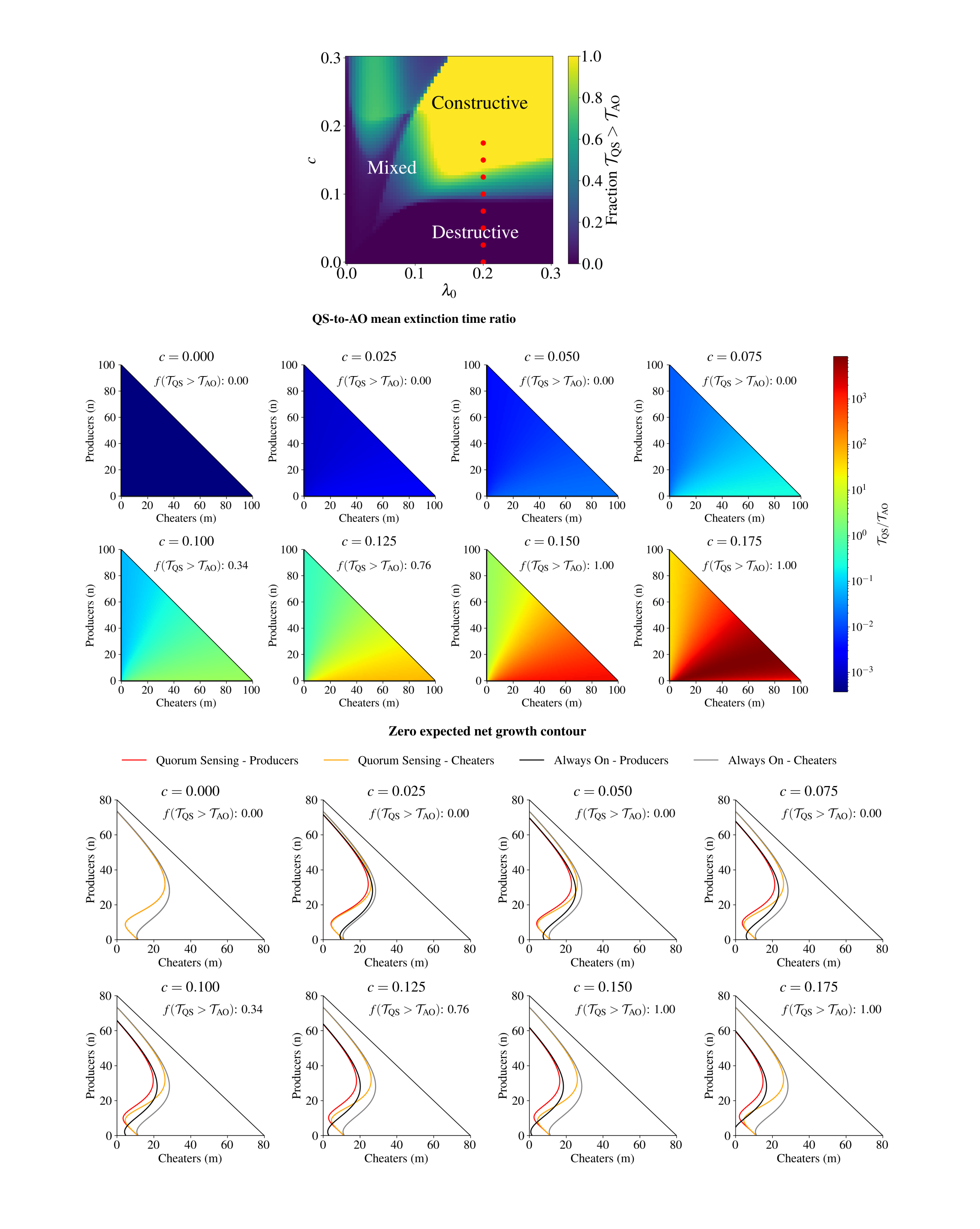

Supplement: S11 Fig — Top row: the red dots shown over the phase diagram indicate the (c, λ0) pairs that we examine in detail. Middle row: the ratio of QS mean extinction time to AO mean extinction time (TQS/TAO) with the indicated values of c. The fraction of (n, m) pairs where (TQS>TAO) is indicated as f(TQS>TAO). Bottom row: the zero expected net growth contour contours for QS producers (red), QS cheaters (orange), AO producers (black), and AO cheaters (grey). At λ0 = 0.075 the diagonal zero expected net growth contour contour near the origin appears for QS, and moves further from the origin as λ0 increases from there. See S2 Table for parameter values. (TIFF) [file pcbi.1010292.s011.tiff]

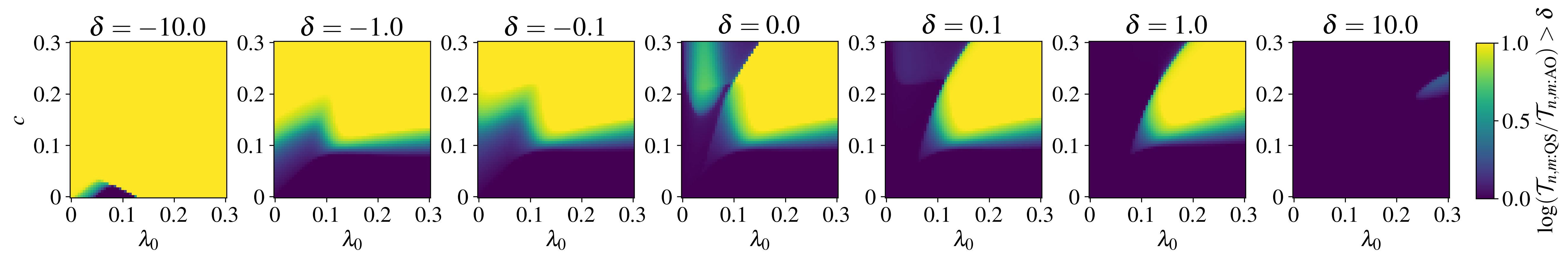

Supplement: S12 Fig — We define a tolerance, δ, so that the above plots display the fraction of (n, m) pairs with log(Tn,m:QS/Tn,m:AO)>δ. Fig 1B corresponds to δ = 0. With slightly larger δ, the region with higher fraction around λ0 = 0.05, c > 0.2 rapidly disappears. This demonstrates that the QS mean extinction times are only slightly larger in this region. (TIFF) [file pcbi.1010292.s012.tiff]

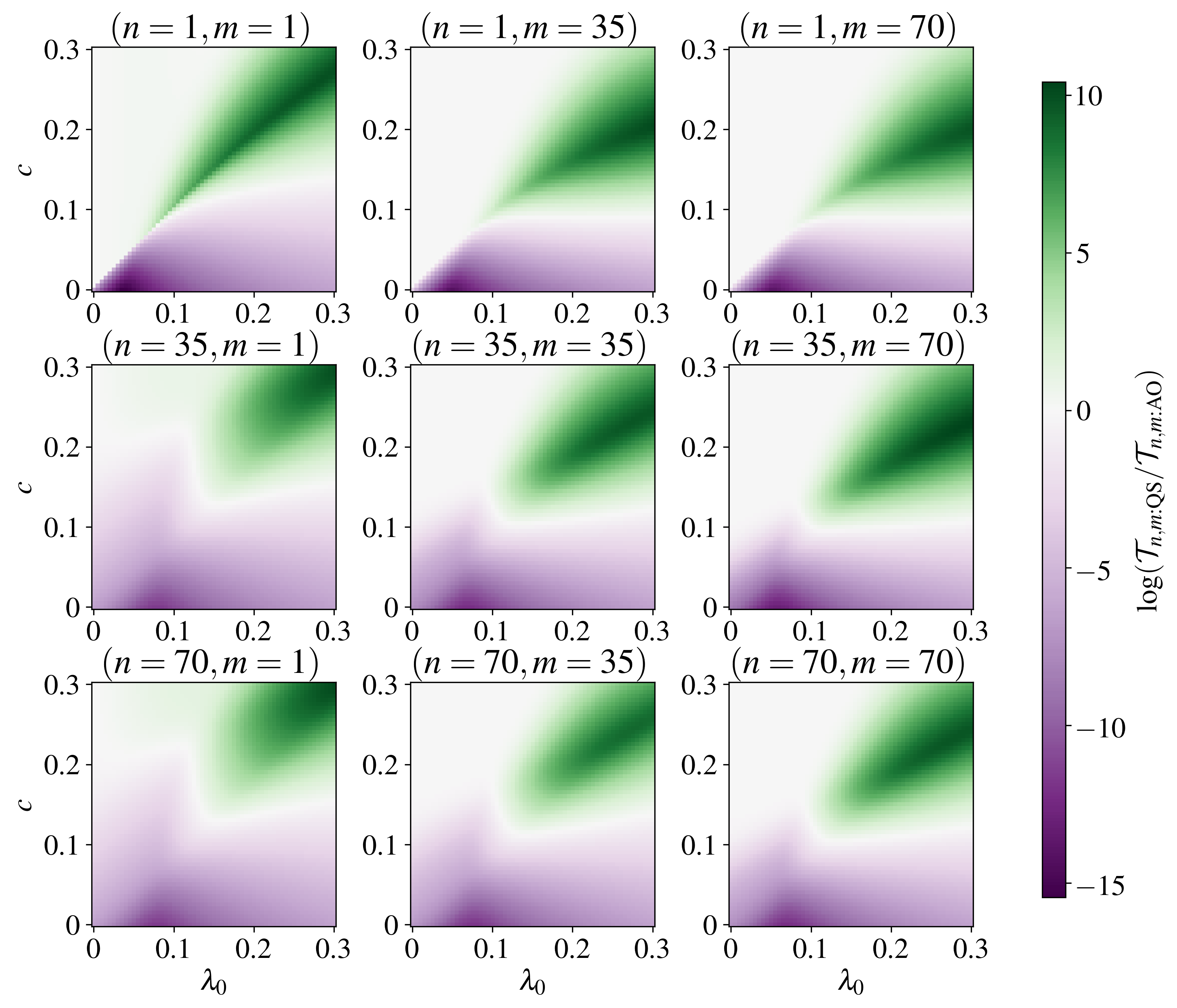

Supplement: S13 Fig — At low c values, Tn,m:QS<Tn,m:AO for all initial conditions, reflecting the destructive region of Fig 1B. With large c and λ0, Tn,m:QS>Tn,m:AO reflecting the constructive region. All other combinations of c and λ0 lead to relatively minor differences between Tn,m:QS and Tn,m:AO. (TIFF) [file pcbi.1010292.s013.tiff]

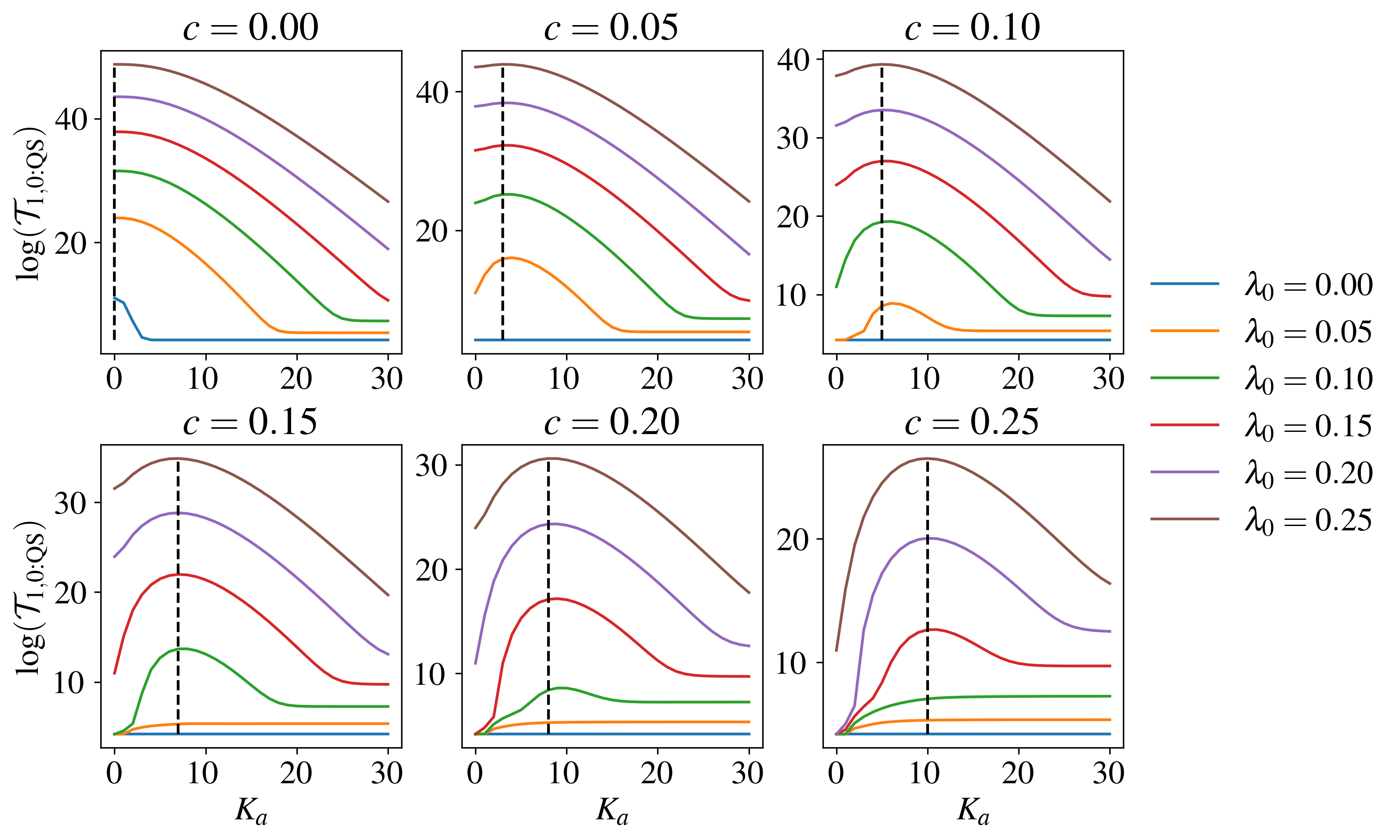

Supplement: S14 Fig — The quantity T1,0:QS represents the ability of a single producer to succeed in establishing a persistent colony in the absence of cheaters. The Ka value that maximizes mean extinction time is weakly dependent on λ0 and increases with larger c, reflecting the larger net benefit of delaying public good activation with higher costs until the public good is more beneficial (determined by the Kg and hg parameters). At c = 0, the AO strategy maximizes the mean extinction time, as there is no cost whatsoever to public good production. Dashed vertical line: optimal value of Ka when λ0 = 0.25. (TIFF) [file pcbi.1010292.s014.tiff]

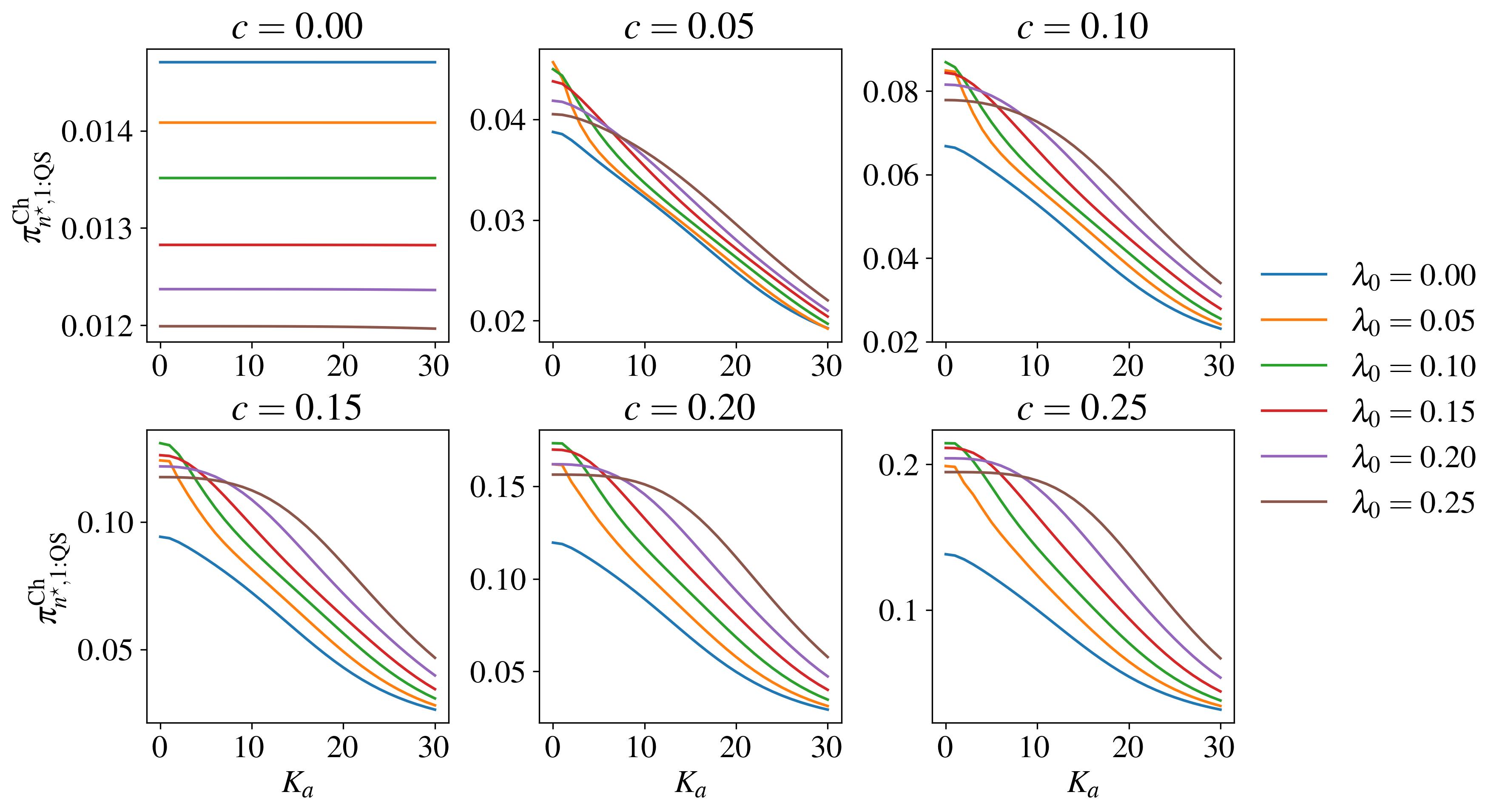

Supplement: S15 Fig — With no cost of public good production (c = 0), Ka has no effect on cheater fixation probability. For all other examined costs, the fixation probability monotonically decreases as a function of Ka for all examined λ0 values. This behavior suggests that the NP strategy, characterized by Ka → ∞, minimizes the cheater fixation probability. (TIFF) [file pcbi.1010292.s015.tiff]

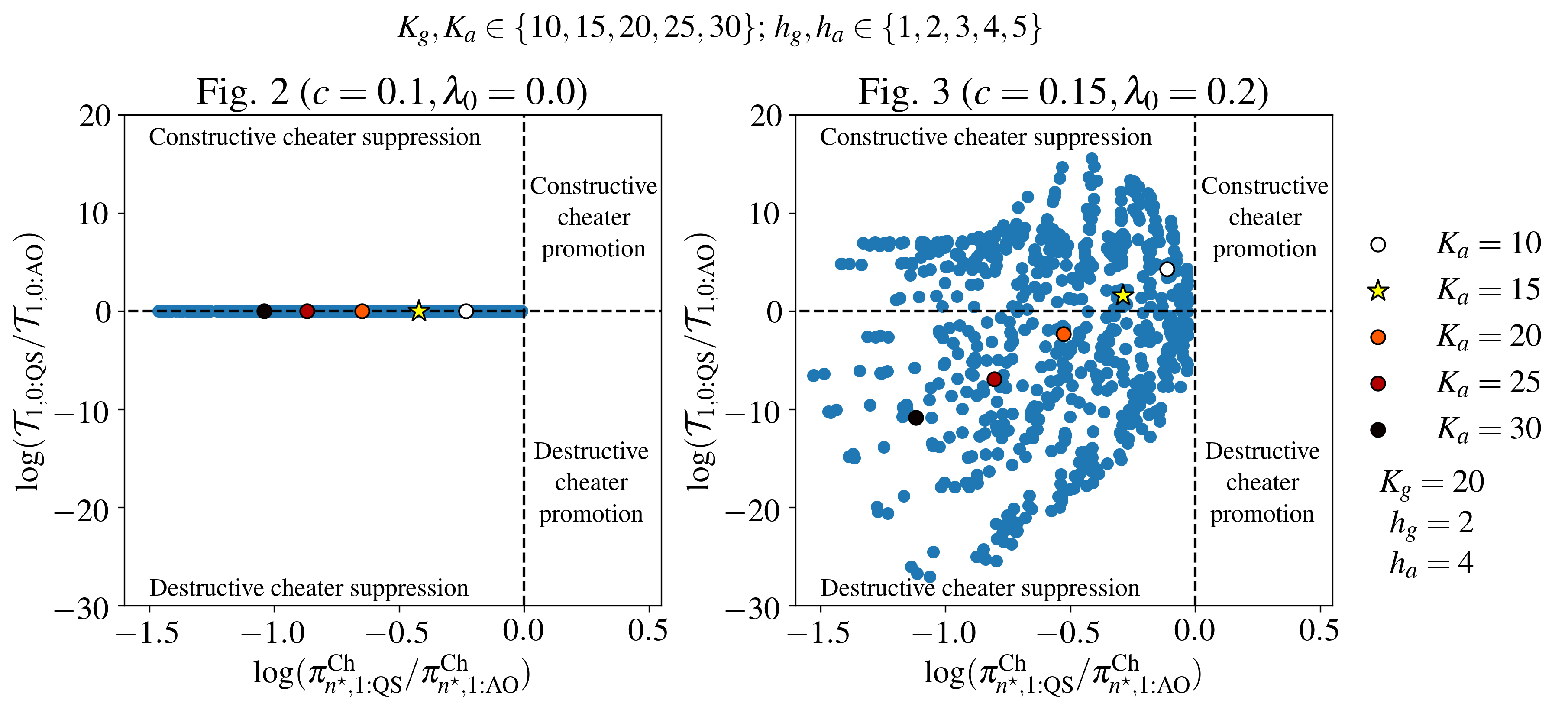

Supplement: S16 Fig — In the left figure, increasing Ka has no effect on mean extinction time, so that loss of public good production altogether (Ka → ∞) minimizes cheater fixation probability compared with AO. Here, we calculated the log-ratio of mean extinction times, log(T1,0:QS/T1,0:AO), and the log-ratio of cheater fixation probabilities, log(πn⋆,1:QSCh/πn⋆,1:AOCh), for all 625 different combinations of Kg, Ka ∈ {10, 15, 20, 25, 30} and ha, hg ∈ {1, 2, 3, 4, 5}. Each point on the plot represents the results for one of the 625 parameter combinations. We calculated mean extinction times for n = 1 producers and m = 0 cheaters, corresponding to the case where a single producer is colonizing an otherwise empty region of space. We calculated cheater fixation probabilities for n⋆=round(λ0+g-cμ) producers and m = 1 cheater, the relevant initial population composition for the case of a single cheater arising by mutation in a population or producers. The highlighted points show how varying Ka alone affects the results (Ka = 15, used in Figs 2 and 3, is indicated by a star), with Kg, hg, and ha fixed as in Figs 2 and 3. (TIFF) [file pcbi.1010292.s016.tiff]
